# The Histone Methyltransferase DOT1L Is a Functional Component of Estrogen Receptor Alpha Signaling in Ovarian Cancer Cells

**DOI:** 10.3390/cancers11111720

**Published:** 2019-11-04

**Authors:** Annamaria Salvati, Valerio Gigantino, Giovanni Nassa, Giorgio Giurato, Elena Alexandrova, Francesca Rizzo, Roberta Tarallo, Alessandro Weisz

**Affiliations:** 1 Laboratory of Molecular Medicine and Genomics, Department of Medicine, Surgery and Dentistry “Scuola Medica Salernitana”, University of Salerno, 84081 Baronissi (SA), Italy; asalvati@unisa.it (A.S.); vgigantino@unisa.it (V.G.); gnassa@unisa.it (G.N.); ggiurato@unisa.it (G.G.) ealexandrova@unisa.it (E.A.); frizzo@unisa.it (F.R.); 2 Genomix4Life Srl, 84081 Baronissi (SA), Italy

**Keywords:** ovarian cancer, ERα, DOT1L, gene expression, estrogen signaling, targeted therapy

## Abstract

Although a large fraction of high-grade serous epithelial ovarian cancers (OCs) expresses Estrogen Receptor alpha (ERα), anti-estrogen-based therapies are still not widely used against these tumors due to a lack of sufficient evidence. The histone methyltransferase Disruptor of telomeric silencing-1-like (DOT1L), which is a modulator of ERα transcriptional activity in breast cancer, controls chromatin functions involved in tumor initiation and progression and has been proposed as a prognostic OC biomarker. As molecular and clinico-pathological data from TCGA suggest a correlation between ERα and DOT1L expression and OC prognosis, the presence and significance of ERα/DOT1L association was investigated in chemotherapy-sensitive and chemotherapy-resistant ER+ OC cells. RNA sequencing before and after inhibition of these factors showed that their activity is implicated in OC cell proliferation and that they functionally cooperate with each other to control the transcription of genes involved in key cancer cell features, such as the cell cycle, epithelial-mesenchymal transition (EMT), drug metabolism, and cell-to-cell signaling, as well as expression of the ERα gene itself. Together with evidence from loss-of-function genetic screens showing that ERα and DOT1L behave as core fitness factors in OC cells, these results suggest that combined inhibition of their activity might be effective against ERα-expressing, chemotherapy-resistant ovarian tumors.

## 1. Introduction

Ovarian cancer (OC) represents a heterogeneous tumor, traditionally classified according to histologic and differentiation grade features [1]. Among OCs, high-grade serous ovarian cancer (HGSOC) represents the most aggressive and lethal form of epithelial ovarian cancer and, although responding among others to platinum-based therapies, the majority of patients relapse after acquiring resistance to first-line treatment [2]. Estrogen receptors (ERs) are expressed in several OC histotypes, with high expression in serous ones, but endocrine therapy has been used with modest and variable results in the treatment of OC [3] sometimes due to the expression of both ERα and ERβ receptor subtypes, which behave in opposite ways after anti-estrogen administration. Thus, targeting specific ER subtypes might help to identify personalized therapeutic approaches and improve survival [4,5,6]. ERα is expressed in more than 50% of OCs and in approximately 80% of HGSOC, where its expression is associated with a poor prognosis [7,8]. Therefore, ERα is worth investigating in OC, given its ability to promote cell proliferation and platinum-resistance [9,10]. A direct action of estrogen in OC growth, metastasis, and progression mediated by ERα has been demonstrated to occur via specific pathways, such as VEGF and MAPK signaling [3]. Moreover, ERα has been implicated as a promoter of metastasis in HGSOC through its involvement in lymphovascular space invasion [7]. 

Epigenetic mechanisms, such as histone modifications and DNA methylation, have emerged as contributing factors to carcinogenesis by playing a pivotal role in regulating the malignant phenotype. Therefore, it represents valuable therapeutic targets [11]. The Disruptor of telomeric silencing-1-like (DOT1L) is the only known histone methyl transferase capable of H3K79 mono-methylation, di-methylation, and tri-methylation associated with active gene transcription [12]. One such modification is thought to be implicated in the de-regulation of genes controlling cancer cell behaviors. DOT1L has been implicated in several chromatin-related functions, including gene transcription and DNA repair, which are involved in the initiation and progression of leukemia and solid tumors [13,14,15], and to represent a potential prognostic and therapeutic marker in OC [16,17]. Furthermore, it was recently demonstrated that this enzyme acts as a key modulator of ERα transcriptional activity in hormone-responsive breast cancer [18]. Starting from these results, the aim of the present study was to search for and investigate functional relationships between ERα and DOT1L in PEO1 and PEO4 HGSOC cells, which are models representing the tumor at its first recurrence and after a second relapse due to the acquisition of chemo-resistance, respectively, and expressing both ERα and DOT1L at appreciable levels. The effects of anti-estrogen treatment on the cell transcriptome and functions demonstrated that ERα signaling is active in both these OC cell lines, where physical association between these two proteins in the cell nucleus was confirmed by co-immunoprecipitation. Inhibition of DOT1L activity with selective inhibitors resulted in cell growth arrest and massive deregulation of cancer-related genomic pathways, including the ERα gene itself and genes involved in cell proliferation, DNA repair, and drug metabolism. Taken together, these results demonstrate that DOT1L is a regulator of ERα activity in estrogen-responsive OCs and an effective drug target for novel therapeutic approaches against these tumors. The results shown in this case suggest that combined ERα and DOT1L inhibition could provide an effective way to treat ERα+ chemotherapy-resistant tumors.

## 2. Results

### 2.1. Characterization of ERα Expressing HGSOC Tumors and Cell Lines

**Figure 1:**
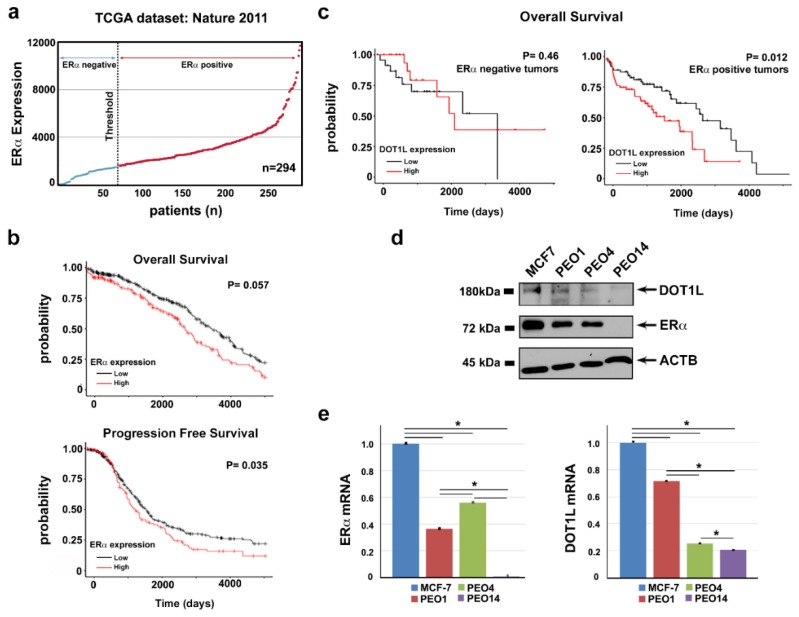
ERα and DOT1L expression in HGSOC tumors. (**a**) ERα mRNA expression in HGSOC from a TCGA cohort. The first quartile, relative to ERα expression level in the tumor, was set as threshold for ERα expression. (**b**) Kaplan-Meier curves of overall survival and progression-free survival for HGSOC patients with respect to an ERα mRNA expression level (low expression: values below first quartile, high expression: values above third quartile). (**c**) Kaplan-Meier curves showing the overall survival relative to DOT1L mRNA expression level (low expression: values below the first quartile, high expression: values above the third quartile) in ERα-negative (left) and ERα-positive (right) HGSOC tumors. Western blots (**d**) and RT-qPCR (**e**) of ERα and DOT1L expression levels in PEO1, PEO4, and PEO14 ovarian cancer cells compared to MCF-7 breast cancer cells. Data are presented as the mean ± SD of three independent replicate RTqPCR assays (* *p* ≤ 0.05).

A correlation between OC prognosis and the presence of ERα has been recurrently considered but is still debated. Analyzing TCGA OC expression profiling datasets [19], we observed that, among 294 HGSOC patients included in this dataset, ERα was highly expressed in 75% of cases (Figure 1a). Considering instead low- ERα and high-ERα mRNA expressing OCs, higher ERα expression results were associated with worse overall and progression-free survival probabilities (Figure 1b). On the other hand, overall survival is significantly impaired in ERα-positive patients who show high DOT1L expression (Figure 1c), which suggests that a combined activity of both these factors might lead to a more aggressive behavior of OC cells, by possibly acting on common targets, as demonstrated previously in breast cancer [18].

**Figure 2:**
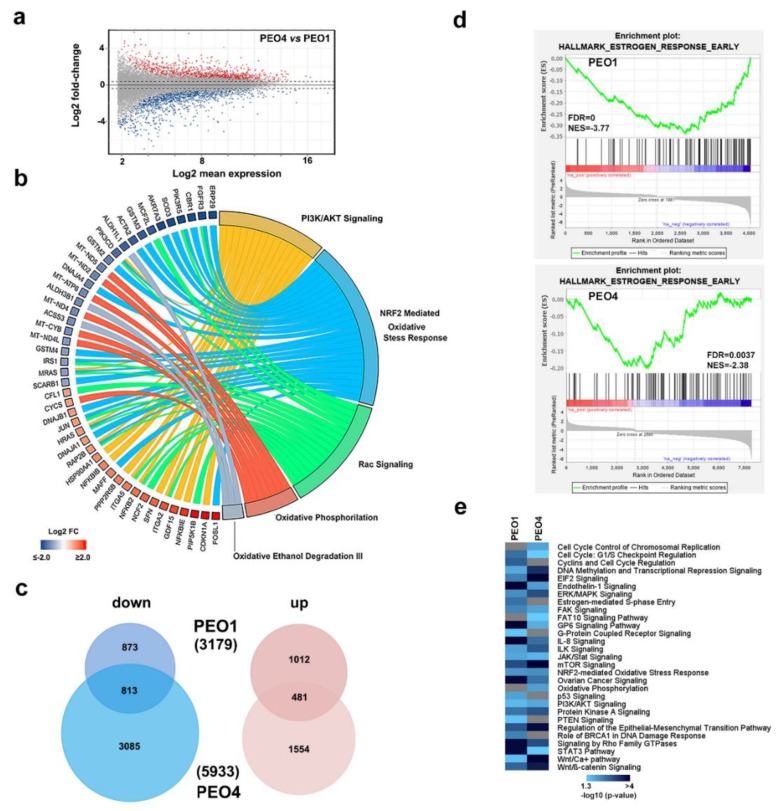
ERα expressing ovarian cancer cell characterization. (**a**) MA plot from RNA-Seq data showing transcriptome differences between PEO4 and PEO1 cells. Sequencing libraries were prepared from three independent biological replicates. (**b**) Circos plot showing transcripts over- (red) and under- (blue) expressed in PEO4 respect to PEO1 cells and influencing the indicated pathways. The length of the arks is proportional to the number of differentially expressed genes belonging to that pathway. Data derived from the list of statistically over-represented (*p*-value ≤ 0.01) pathways are selected based on differentially expressed genes (Log2 FC PEO4 vs. PEO1). (**c**) Venn diagrams showing the number of transcripts down- (light blue) or up- (pink) regulated by ICI (100 nM) in either PEO1 or PEO4 cells. 1.5 fold-change cut-off was selected as the threshold to identify differentially expressed genes. (**d**) Gene set enrichment analysis (GSEA) showing the Early Estrogen Response GO term highlighted by ICI-modulated genes in PEO1 and PEO4 cells. Negative Normalized Enrichment Score (NES) indicate that down-regulated genes are over-represented. (**e**) Statistically significant signaling pathways revealed by differentially expressed genes by ICI (100 nM) in PEO1 and PEO4 cells. Gray bars indicate a lack of statistically significant enrichment.

To investigate this possibility, we adopted as experimental model of two cell lines, PEO1 and PEO4, isolated from the same individual and representing two stages of the disease, which are the first recurrence stage and the chemo-resistance stage [20,21]. Behan et al. [22] recently investigated the importance of tumor molecular features in guiding the prioritization of cancer therapeutic targets on 324 cell lines of different cancer types, including PEO1 and PEO4, searching for genes required for cancer cell fitness (defined as cell growth and viability). ESR1, which represented the gene coding for ERα protein, resulted in a key gene in both these OC cell lines, since its inactivation caused a loss of fitness, which indicated that this receptor is a favorable therapeutic target in these cells (data not shown). Protein and mRNA expression assays confirmed co-expression of both ERα and DOT1L in PEO cells, even though their level was slightly different between the two, and lower when compared to those of breast cancer MCF7 cells used for comparison (Figure 1d,e). Comparative transcriptome analysis, which is performed by RNA-Seq, led to the identification of a consistent number of differentially expressed genes in the two cell lines (Figure 2a and Supplementary Table S1A) revealing that activity of drug resistance pathways is significantly different in the two cell lines (Figure 2b).

Among these, the NRF2-mediated oxidative stress response, together with other oxidative stress pathways, has been implicated in OC resistance to chemotherapeutics [23]. NRF2 has been recently demonstrated to be a molecular partner of ERα in OC, where its co-expression with the receptor impacts patient survival [24]. Although NRF2 mRNA itself did not show significant differences in expression between the two cells, other genes on the pathway were affected (light blue arks in Figure 2b), which led to a prediction of an upregulation of this pathway in PEO4 compared to PEO1 cells (z-score 1.826, data not shown). Moreover, many of the genes up-regulated in PEO4 cells participate in PI3K/AKT signaling, which is particularly relevant in OC. Several inhibitors of this pathway have been tested against these tumors [25,26]. Despite the gene expression differences between these cell lines derived from the same patient, they responded similarly to anti-estrogens, as shown by gene expression profiling performed after ICI 182,780 (ICI, fulvestrant) treatment, known to induce appreciable effects in these cells [9]. A functional analysis performed on all genes regulated by ICI in both cell lines (Figure 2c and Supplementary Table S1B,C) revealed multiple pathways similarly affected by the drug in both cell lines. In particular, Gene Set Enrichment Analysis (GSEA) revealed statistically significant hallmark GO terms over-represented after ICI treatment in both PEO1 and PEO4 cells. As shown in Figure 2d, for example, the Early Estrogen Response GO term was significantly represented in both cell lines. Although not all genes of this pathway were regulated to the same extent in both cell lines, the global effect was, in both cases, due mainly to down-regulated genes, as demonstrated by the negative Normalized Enriched Score (NES -3.77 and -2.38 for PEO1 and PEO4 respectively), even though these results are more evident in PEO1. Moreover, Figure 2e shows the functional pathways similarly enriched in both cell lines and those specific for each individual cell line. Among them, many are linked to known effects of estrogen in target cells, such as those involved in cell cycle checkpoints (commonly enriched), cell cycle control of chromosomal replication (PEO4-enriched), cyclin regulation (PEO1-enriched), and estrogen-mediated signaling and S-phase entry (PEO1-enriched), which suggests that inhibition of ERα activity might interfere with cell cycle completion and cell proliferation in both of these OC cell lines, even if specifically acting on certain components.

### 2.2. DOT1L Inhibition Causes Growth Arrest and Transcriptome Deregulation in OC Cells

**Figure 3:**
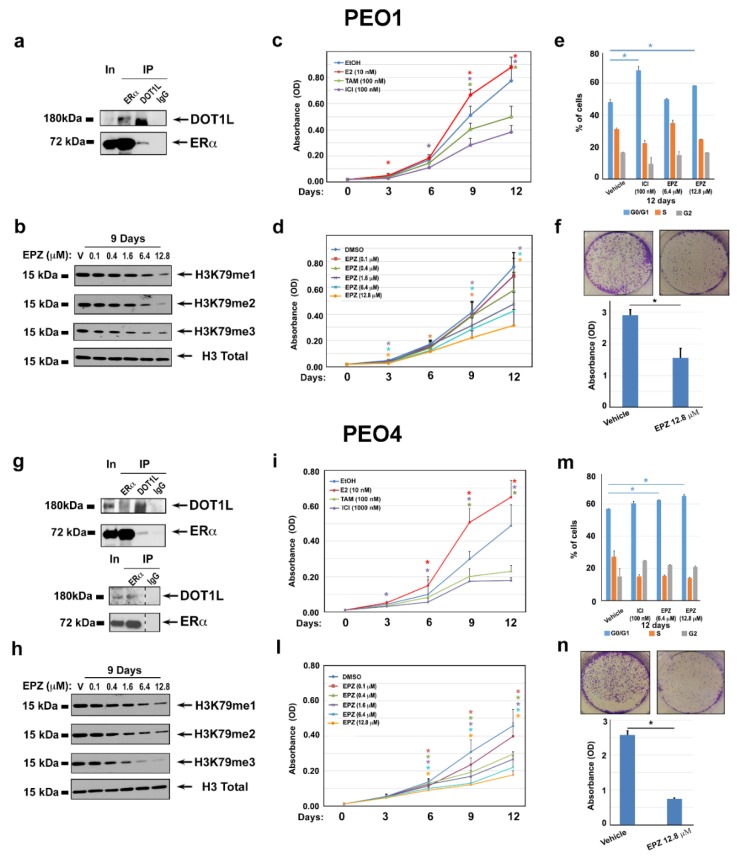
Functional effects of ERα/DOT1L complex disruption by pharmacological inhibition. ERα and DOT1L co-immunoprecipitation in PEO1 (**a**) and PEO4 (**g**) nuclear extracts (the lower panel in g shows the results of an independent ERα IP test). WB analysis performed in PEO1 (**b**) and PEO4 (**h**) cells showing the effect on H3K79me1, me2, and me3 compared to total H3 after nine days of treatment with vehicle (V, DMSO) or increasing concentrations of EPZ004777 (EPZ). The MTT assay performed in the presence of estrogen (17β-estradiol, E2) (10 nM), antiestrogens Tamoxifen (TAM) (100 nM), and ICI 182 780 (ICI) (100 nM) (**c** and **i**), or increasing concentrations of EPZ (**d** and **l**). Vehicles (EtOH or DMSO) were used as negative controls. Bar plots of PI-stained PEO1 (**e**) and PEO4 (**m**) cells showing percentages of G1, S, and G2/M cells after 12 days of treatment with ICI, EPZ, or the control vehicle, as indicated. A colony formation assay performed in triplicate on PEO1 (**f**) and PEO4 (**n**) cell lines treated with vehicle (V, DMSO) or 12.8 μM of EPZ for 12 days. Colonies were visualized by crystal violet staining. Error bars represent the mean of replicate values ± SD (* *p* ≤ 0.05).

Based on the evidence that DOT1L is a co-factor and upstream regulator of ERα in breast cancer [18], we verified the possibility that ERα associates with DOT1L in PEO cells by co-immunoprecipitation experiments. Results shown in Figure 3a,g reveal that this is the case, with the dose-dependent reduction of H3K79 mono-methylation, di-methylation, and tri-methylation in response to a DOT1L blockade with the specific inhibitor EPZ004777 (EPZ, Figure 3b,h) demonstrating that this enzyme is required to maintain the levels of H3K79 methylation in both cell lines. 

Then, to assess the effects of ERα and DOT1L inhibition on OC cell proliferation, MTT assays were performed before and after treatment with anti-estrogens (tamoxifen, TAM, and fulvestrant, ICI) and increasing concentrations of EPZ. The results obtained confirmed the responsiveness of both PEO cell lines to the mitogenic effects of estrogen, which was demonstrated by the ability of anti-estrogens to inhibit cell proliferation (Figure 3c,i). On the other hand, EPZ was able to reduce OC cell proliferation in a dose-dependent and time-dependent manner (Figure 3d,l), with the maximum effect observed after 12 days with 6.4 to 12.8 µM EPZ. Cell cycle analysis before and after treatment with either of these compounds showed that this is due to cell cycle inhibition, which was revealed by an increase of G0/G1 cells accompanied by a specular reduction of S-G2 cells after ICI or EPZ (Figure 3e,m). While EPZ treatment determined a significant reduction of PEO1 and PEO4 colonies formation (Figure 3f,n), no marked effect was observed on apoptosis after cell exposure to the inhibitor for up to 12 days, which caused only a minimal increase of caspase cleavage and appearance of sub-G1 cells (data not shown). The same effects on H3K79 methylation and cell proliferation was observed after a DOT1L blockade with other inhibitors, such as EPZ5676 and SGC (Supplementary Figure S1A–G). Treatment of ERα-negative PEO14 cells with the DOT1L inhibitor resulted in a reduction of H3K79 methylation similar to what was observed in PEO1 and PEO4 cells, while no significant effects could be observed on the cell cycle and cell proliferation (Supplementary Figure S2A,B).

**Figure 4:**
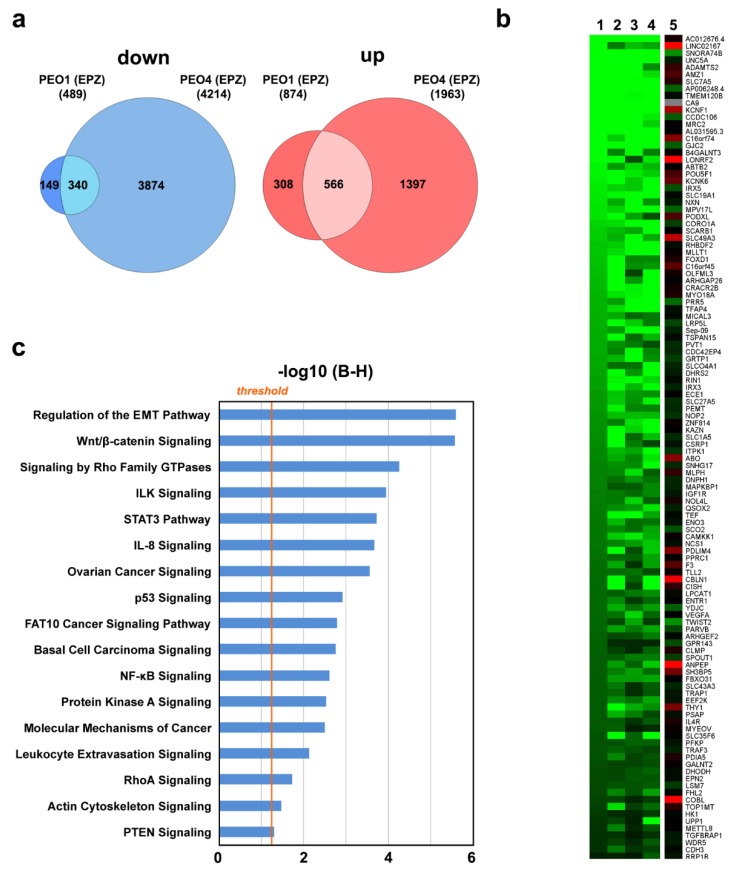
Effects of DOT1L pharmacological inhibition on OC cell transcriptome. (**a**) Venn diagrams showing down- (left) and up- (right) regulated transcripts after EPZ (6.4 μM) treatment in both PEO1 and PEO4 cells. RNA-Seq was performed in biological triplicates. (**b**) The heatmap showing transcripts regulated by both ICI (100 nM) or EPZ (EPZ, 6.4 μM) in both PEO1 and PEO4 cells, compared to EPZ-treated ERα-negative PEO14 cells. (**c**) Graphic representation of statistically significant pathways, identified by IPA analysis, considering genes responding to DOT1L and ERα inhibition with EPZ and ICI, respectively, in PEO1 and PEO4, but not PEO14, cells. The straight orange line marks the Benjamini Hochberg *p*-value (B–H) threshold (0.05).

Since H3K79 methylation by DOT1L is directly coupled to gene transcription [27], we then focused our attention on deregulation of the OC cell transcriptome by DOT1L inhibition (Figure 4). A comparison of differentially expressed genes after treatment with EPZ revealed 340 transcripts down-regulated and 566 up-regulated in common between PEO1 and PEO4 cells (Figure 4a and Supplementary Table S1D,E). To consider the biological effects mediated by both ERα and DOT1L, we discarded the genes showing the same response to EPZ in the ERα-negative OC cell line PEO14, where DOT1L is expressed and its inhibition with EPZ results in a dose-dependent decrease of H3K79 methylation and modulation of several genes, including some that responded to the inhibitor in either PEO1 or PEO4 cells (Supplementary Table S1F and Supplementary Figure S2).

The heatmap of Figure 4b summarizes the results relative to 194 genes that respond in the same way to ERα and DOT1L inhibition with ICI and EPZ, respectively, in PEO1 and PEO4 but not PEO14 cells, where they were either not affected by these treatments, showed statistically insignificant changes, or were the opposite compared to what was observed in the other two cell lines. These genes belong to pathways functionally relevant in OCs, such as regulation of invasiveness/migration (EMT, Wnt/β-catenin, Rho, ILK) and control of key signaling cascades (STAT3, IL-8, p53, FAT10, NF-kB) (Figure 4c).

### 2.3. DOT1L is a Transcriptional Co-Regulator of ERα in OC Cells

**Figure 5:**
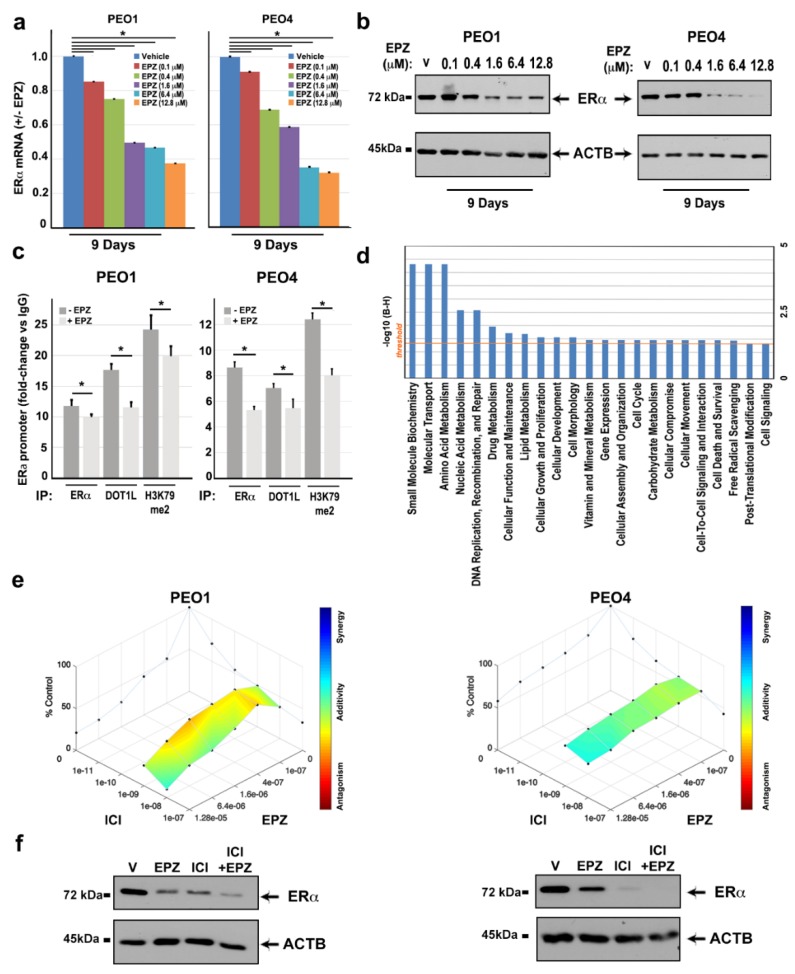
Effects of EPZ on ERα-DOT1L co-localization and transcriptional regulation of target genes with ERE motifs and anti-proliferative activity in combination with anti-estrogen compounds. RT-qPCR (**a**) and immunoblotting (**b**) analysis show the effects of EPZ, with respect to Vehicle (V, DMSO), on ERα mRNA and protein. Error bars represent the mean of triplicate values ± SD (* *p* ≤0.05). (**c**) ChIP–qPCR analysis of ERα, DOT1L, and H3K79me2 localization in the ERα promoter region before and after nine days of treatment with EPZ (6.4 μM). Data are relative to triplicate measurements, ± SD (* *p* ≤ 0.05). (**d**) IPA Pathway analysis on ERE motif-containing genes downregulated by ICI and EPZ in both PEO1 and PEO4 cells. The straight orange line marks the Benjamini Hochberg *p*-value (B-H) threshold (0.05). (**e**) D-R Lowe graph showing the effects of drug combination between two fixed doses of ICI (1 and 10 nM) and increasing EPZ concentrations after nine days of treatment. (**f**) Western blot analysis of ERα protein levels after treatment with ICI (10 nM) and EPZ (6.4 μM) alone or in combination. Vehicle (V, DMSO) was used as a control.

As previously shown in breast cancer [18], DOT1L inhibition resulted in a dose-dependent inhibition of ESR1 gene expression in both cell lines, as demonstrated by a decrease of both ERα mRNA (Figure 5a) and protein (Figure 5b) levels upon EPZ treatment. This is a result confirmed with the other DOT1L inhibitors EPZ5676 and SGC (Supplementary Figure S3a,b). 

To investigate whether the effects of DOT1L inhibition on ERα gene expression are mediated by a direct role of this enzyme on ESR1 gene transcription, we analyzed a regulatory region of this gene promoter region for the presence of ERα, DOT1L, and the active transcription marker H3K79me2 by ChIP–qPCR. Results shown in Figure 5c demonstrate that this is the case, indicating that, similarly to luminal-like breast cancer cells, the two factors associate on the chromatin and are likely to cooperate with each other for regulating estrogen target genes, as demonstrated for the ESR1 gene itself. EPZ administration caused a reduction of ERα, and, consequently, DOT1L binding to this promoter (Figure 5c), which was accompanied by its inhibition. This was demonstrated by a decrease of H3K4me3 and accumulation of H3K27me3 epigenetic marks (Supplementary Figure S4). These responses to DOT1L inhibition are more evident in PEO4 cells, which is a result in line with the more evident responses to EPZ of these cells compared to PEO1. When combined, these results further support the possibility of a functional cooperation between DOT1L and ERα to control ESR1 gene transcription, demonstrated by the reduction of active transcription marks (H3K79me2 and H3K4me3) and increase repressive ones (H3K27me3) within this gene promoter upon DOT1L inhibition, which leads to ERα downregulation. Considering that this mechanism of action of DOT1L is likely to be in place on a number of other genetic elements responsive to ERα, we identified ERE (Estrogen-Response-Elements) motifs in the regulatory regions of 50% of genes inhibited by both ICI and EPZ in PEO1 and PEO4 cells (Supplementary Table S2). Functional analysis, which is performed when considering the consequences of the repression of these genes consequent to ERα and DOT1L blockade (Figure 5d), revealed that the DOT1L/ERα complex appears to affect not only cell growth, proliferation, death and survival, as well as lipid, carbohydrate, vitamin, drug metabolism, DNA recombination and repair, and cell morphology and cell movement. This indicates that targeting this complex might be useful not only to overcome cell proliferation but also metabolic tuning and drug resistance in OC. To verify this possibility, a set of ERE-containing promoters were selected among EPZ-downregulated genes and tested for ERα and DOT1L binding. Results show ERα occupancy in HOXB3, TGM1, TWIST2, and WT1 gene promoters, which are all relevant for ovarian cancer, and DOT1L co-binding with ERα only in the TWIST2 and WT1 promoter regions (Figure S5). Since all these genes are down-regulated by EPZ, this result indicates that the DOT1L blockade exerts an effect on gene activity via multiple mechanisms, including a direct effect on ERα/DOT1L complexes and more complex effects on ERα activity, which might include ERα downregulation.

Simultaneous inhibition of ERα and DOT1L by a combination of ICI and EPZ results in an additive effect of the two drugs on cell proliferation and ERα protein levels. This was achieved by evaluating cell proliferation after nine days of treatment with two fixed doses of ICI (10^−8^ and 10^−9^ M) and increasing concentrations of EPZ. Results show an additive effect of the combined treatment on cell proliferation and ERα levels (Figure 5e,f), which suggests that both drugs appear to act on a common target. Consistent with the other data reported above, this result suggests that it is possible that both drugs act via the ERα/DOT1L complex.

A recent genome-wide CRISPR–Cas9 loss-of-function genetic screen to identify genes essential for proliferation and survival of cancer cells (indicated as “Achille” genes) [28] showed that DOT1L, whose expression correlates with a worse clinical behavior in OC patients (Supplementary Figure S6A) is required for optimal survival in about 60% of all investigated OC cells (Supplementary Figure S6B). On the other hand, among the 37 OC cell lines investigated in that study, ERα had a significant negative Dependency Score (≤−0,1), which indicates that this factor plays an important functional role in the maintenance of OC cells’ viability, in four (COV413A, SNU8, JHOS2, SKOV3) out of six cell lines expressing it at appreciable levels (ESR1 mRNA transcripts per million ≥ 0.5). This confirms what was observed here experimentally and computationally, by analysis of cancer fitness genes data from Behan et al. [22], in ER + PEO1 and PEO4 cells. 

Taken together, these results indicate that DOT1L inhibition can be effective against a sizeable fraction of ovarian tumors.

## 3. Discussion

ERα is a well-known cancer-associated gene, target of anti-estrogen based therapies in breast cancer, and expressed in a large OC fraction, where its prognostic value is still debated. OCs represent a highly heterogeneous group of cancers, with different etiology, phenotypes, molecular biology, and clinico-pathological features, including in the prognosis. Epithelial ovarian cancers (EOCs) represent the vast majority of these tumors, and, among them, HGSOCs are the most aggressive and account for almost 75% of all EOCs [29]. As a matter of fact, OC remains the most lethal gynecologic malignancy due to late stage diagnosis, recurrence, and a low response rate to current treatments. While the gold standard for OC therapy has been based on a primary debulking/staging surgery, along with platinum-based chemotherapy, emerging strategies have focused on targeting single factors and pathways intrinsically involved in tumor growth and metastasis [30,31,32]. Molecular characterization has provided evidence of the most recurrent genomic alterations in OC and their use for diagnosis and treatment, which allows the development and application of therapeutic approaches targeting angiogenetic (VEGF inhibitors), DNA repair (PARP inhibitors), and other signaling (PI3K and AKT inhibitors) pathways [33,34].

Despite active ERα-mediated estrogen signaling having been observed in OC [9,35], which makes anti-estrogen therapy an attractive premise, this last process has been restricted so far to chemo-resistant tumors, where discordant responses following tamoxifen or ICI (fulvestrant) administration have been reported [36,37]. This may be a consequence of the tissue specificity of these compounds, explained, in part, by differences in the repertoire of ERα co-regulators in the ovary when compared to the best characterized breast tissue. This determines divergent and poorly overlapping effects of the receptor on gene modulation in the two tissues [35]. The molecular mechanisms by which ERα promotes OC cell growth and other key cellular functions are still unclear [35,38]. These involve in this case, as in other tissues, chromatin-associated multiprotein complexes endowed with transcription regulatory functions that act with ERα in its target sites within gene promoters and other regulatory genetic elements to convey estrogen signaling to the genome [39,40]. In breast cancer, several proteins functioning as epigenetic chromatin modifiers have been demonstrated to be part of these complexes, where they are responsible for the chromatin modification/remodeling steps essential to allow ERα-dependent gene regulation [18,41,42]. These include the histone methyltransferase DOT1L, which has been suggested to be of prognostic value in OC since it enhances cell cycle progression and drug resistance by acting as a transcriptional co-regulator of G1-phase genes [16] and for transcription factors acting on multi-drug resistance genes [17]. 

We report in this paper how inhibition of estrogen signaling by targeting this upstream ERα co-factor and regulator may represent an effective therapeutic approach against ERα-expressing OCs. Recently, it has been demonstrated that silencing ERα expression and activity through the use of epigenetically-acting compounds is a good option in breast cancer to overcome resistance to endocrine therapy [18,41]. In this study, we show that the same approach may be effective in reinforcing anti-estrogen effects in ERα-expressing OCs, which are either sensitive or refractory to standard therapies. Starting from the observation that ERα and DOT1L co-expression is associated with poor survival probability in OC, we demonstrated that interfering with ERα/DOT1L complex activity by either ERα or DOT1L inhibition with selective antagonists results in a dose-dependent reduction of OC cell proliferation, which possibly occurs through a G1 phase arrest and is mediated by significant changes in the cell transcriptome. Mechanistically, this might be explained by the fact that selective inhibition of either protein interferes with ERα and DOT1L co-recruitment onto regulatory elements of ESR1 gene, and H3K79 methylation. The finding that several EPZ-regulated genes show ER-binding motifs (EREs) within their promoter region further support the possibility of an effect of DOT1L on ERα signaling, substantiated by evidence of ERα/DOT1L co-occupancy of some of these promoters.

In conclusion, the results described in this study provide evidence of a functional cooperation between ERα and DOT1L, acting at the transcriptional level to modulate the expression of genes involved in OC cell proliferation and other key cellular functions. Since specific inhibitors of the two proteins show additive effects on OC cell growth, it is possible to assume that dual inhibition of estrogen signaling by simultaneous blockade of ERα and DOT1L may represent a way, worth exploring further, to improve the response and survival of patients suffering with ERα-positive OC, where endocrine therapy usually shows only moderate responses. DOT1L expression correlates with a worse clinical behavior of OCs and genome-wide in vitro genetic screens by the CRIPSR-Cas9 gene ‘knock-down’ revealing that this gene is important for proliferation and survival of >60% of the OC cell lines tested [28]. The results reported in this study indicate that DOT1L may represent an actionable drug target in OCs, which suggests the need for further in-depth mechanistic studies in vitro, accompanied by validation tests with patient-derived cells and xenografts, to further clarify this potentially promising new way to treat these aggressive cancers.

## 4. Materials and Methods 

### 4.1. TCGA and Cell Lines Data Analysis

Data from the TCGA Ovarian Serous Cystadenocarcinoma [19] dataset were retrieved using the cBioPortal and survival analysis was performed using the survminer R package. Overall and progression-free survival curves were generated with the Kaplan Meier Plotter (kmplot.com). To determine groups presenting low-expression and high-expression of ERα and DOT1L, quartile filtering was applied. In detail, cases with expression levels below the first quartile were included in the ‘low expressing’ group, while those above the third quartile were included in the ‘high expressing’ one.

CRISPR-Cas9 gene knock-down data have been downloaded from DepMap portal (https://depmap.org) and from the Project Score website (https://score.depmap.sanger.ac.uk).

### 4.2. Cell Lines

Human ovarian cell lines ERα-positive PEO1 (ECACC 10032308) and PEO4 (ECACC 10032309) and ERα-negative PEO14 (ECACC 10032311) were purchased from the American European Collection of Authenticated Cell Cultures (ECACC). All cell lines were isolated from peritoneal ascites of serous ovarian adenocarcinomas and carry TP53 mutations [43]. PEO1 cells display a mutation in the BRCA2 gene, which is reverted by a secondary mutation in PEO4 [44]. All cell lines were cultured in RPMI 1640 medium (Euroclone, Milan, Italy) supplemented with 10% FBS (HyClone, Milan, Italy) and antibiotics: 100 U/ml penicillin, 100 mg/mL streptomycin, and 250 ng/mL Amfotericin-B. Cell lines were routinely tested for *Mycoplasma* contamination with a PCR mycoplasma detection kit (Abm, Richmond, BC, Canada).

### 4.3. Antibodies and Compounds

The following antibodies were used for immunoprecipitation and Western blot analyses: C-terminal anti-ERα (F-10 sc-8002, Santa Cruz Biotechnology, Dallas, Texas), rabbit anti-estrogen Receptor Alpha (ab32063, Abcam, Cambridge, UK), rabbit polyclonal anti-DOT1L (A300-953A, Bethyl Laboratories, Montgomery, Alabama), β-actin (A1978, Sigma Aldrich, Milan, Italy), Rabbit anti-KMT4/DOT1L (ab72454), anti-Histone H3, total, (ab1791), anti-H3K79me1 (ab2886), anti-H3k79me2 (ab3594), anti-H3K79me3 (ab2621), anti-H3K4me3 (ab7766), anti-H3K27me3 (ab24684) from Abcam, anti-Rabbit IgG Isotype Control (31235, Thermo-Fisher), and the anti-Mouse IgG antibody (RM104, Aurogene, Rome, Italy).

Cells were treated with the following compounds: DOT1L inhibitors EPZ004777 (S7353), Pinometostat (EPZ5676)(S7062), SGC0946 (S7079), all from Selleckchem, and with 4-hydroxytamoxifen (4-OHT) (H7904, Sigma-Aldrich), Fulvestrant (ICI 182,780) (I4409, Sigma-Aldrich), and β-estradiol (E887-5G, Sigma-Aldrich) or vehicles (DMSO and/or EtOH), according to different experimental settings.

### 4.4. RNA Extraction and Sequencing

Total RNA was extracted using the standard RNA extraction method with TRIzol (Thermofisher Scientific, Waltham, MA, USA), according to the manufacturer’s instructions. Furthermore, 0.3–1 mL of TRIzol™ Reagent per 1 × 10^5^–10^7^ cells was added directly to the culture dish to lyse the cells by pipetting several times to homogenize. 

Before use, RNA concentration was determined by using Quant-IT RNA Assay Kit-High Sensitivity and a Qubit Fluorometer (Life Technologies, Monza, Italy) and its quality and integrity assessed with the Agilent 4200 Tapestation System (Agilent Technologies, Milan, Italy).

For RNA sequencing, cell lines were treated with 6.4 µM EPZ004777, 10^−8^ M ICI 182,780, and their vehicles for nine days. Indexed libraries were prepared, using 1 μg of total RNA as starting material from biological triplicates, with TruSeq Stranded Total RNA Sample Prep Kit (Illumina Inc., San Diego, CA, USA). Libraries were sequenced (paired-end, 2 × 75 cycles) at a concentration of 1.8 pM/lane on the NextSeq 500 platform (Illumina Inc.). The raw sequence files generated (.fastq files) underwent quality control analysis using FASTQC (http://www.bioinformatics.babraham.ac.uk/projects/fastqc/) and quality-checked reads were then aligned to the human genome (assembly hg19) using a star [45] with the standard parameters. Data were analyzed as previously described [46]. A gene was considered differentially expressed when shown a |Fold-Change| (FC) ≥ 1.5 and an adjusted *p* value (adj-*p*) ≤ 0.05. Raw RNA sequencing data are deposited in the EBI ArrayExpress database (http://www.ebi.ac.uk/arrayexpress) with accession number E-MTAB-8198.

### 4.5. RT-qPCR

One μg of total RNA for each sample was reverse transcribed to cDNA using Random Hexamer (Tetro cDNA Synthesis Kit, Bioline, Memphis, Tennessee). RTqPCRs were performed in triplicate on a Stratagene Mx3005P instrument (Agilent Technologies) using SensiFAST SYBR Lo-ROX kit (Bioline), according to the manufacturer’s instructions. Values were normalized to values relative to RPLP0 mRNA, analyzed in parallel.

The primers used for qPCR are shown below. 

for RPLPO: Forward primer: CCATCAGCACCACAGCTTC 

    Reverse primer: GGCGACCTGGAAGTCCAACT 

for ERα: Forward primer: ACCCTCCATGATCAGGTCCA 

    Reverse primer: CTGGTTCCTGTCCAAGAGCA

for DOT1L: Forward primer: GTTCTACCAGCTACCTCCGAGCGTGCAGC

    Reverse primer: *GCTGCACGCTCGGAGGTAGCTGGTACAAC*

### 4.6. Protein Extraction and Co-Immunoprecipitation

Protein extractions were performed after culturing in a normal growing condition with either vehicle (at the longer time point) or DOT1L inhibitors for 3, 6, 9, or 12 days. Media were refreshed every 3 days. 

For total protein extraction, cells were harvested and lysed in an equal volume with respect to the cell pellet of high salt buffer (Tris-HCl pH 7.5 50 mM, NaCl 500mM, NP40 0.15%, Glycerol 10%, MgCl_2_ 1.5 mM, NaMo_4_ 1mM, and NaF 0.5 M). After incubation on ice for 15 min, samples were centrifuged at 13.000× *g* for 30 min and the supernatant, containing total proteins, was transferred to a new tube and diluted by adding two volumes of low salt buffer with respect to the high salt buffer previously added. 

Nuclear and cytosolic protein extraction was performed as previously described [47]. 

Cells were harvested in ice cold PBS and pellets were suspended in Hypotonic Buffer (200 mM HEPES pH 7.4, 50 mM NaF, 10 mM sodium molybdate, 0.1 mM EDTA, 1 mM PMSF, and 1X protease inhibitor cocktail) to isolate a cytosolic fraction. Then, the nuclear pellets were dissolved in a Nuclear Lysis Buffer (20 mM HEPES pH 7.4, 25% (*v/v*) glycerol, 420 mM NaCl, 1.5 mM MgCl_2_, 0.2 mM EDTA, 1 mM DTT, 1× protease inhibitor cocktail, and 1 mM PMSF), incubated for 30 min at 4 °C and centrifuged for 30 min at 4 °C. Nuclear proteins were then diluted to restore the physiological saline concentration. Protein concentration was established by a Bradford Assay.

For ERα and DOT1L immunoprecipitations, 35 μL of equilibrated Dynabeads M-280 Sheep Anti-Rabbit IgG (Thermo Fisher Scientific) were conjugated overnight at 4 °C, respectively, with 2 μg of anti-ERα, anti-DOT 1L, and a Rabbit IgG Isotype Control. Immunoprecipitation was performed by incubating conjugated beads/antibodies at 4 °C for 1 hour with 500 μg of nuclear protein extracts. After incubation, beads were washed with IPP150 buffer (7.14 mM HEPES pH 7.5, 8.92% glycerol, 150 mM NaCl, 0.54 mM MgCl_2_, 0.07 mM EDTA pH 8, and 1× protease inhibitors) and Wash Buffer (50 mM Tris-HCl pH 7.6, 150 mM NaCl, and 1× protease inhibitors), and suspended in Laemmli buffer.

### 4.7. Histone Extraction 

All cell lines were seeded at a concentration of 10^5^ cells/35 mm plate. PEO1 and PEO4 were incubated for 3, 6, 9, and 12 days with increasing concentrations of EPZ00477 and for 9 days with increasing concentrations of EPZ5676 and SGC or the control vehicle. PEO14 were incubated with different concentrations of EPZ00477 and its vehicle for 12 days. Medium compounds were refreshed every 3 days. 

At the indicated time points, cells were harvested, washed in ice cold PBS, and suspended in Triton Extraction Buffer (TEB: PBS containing 0.5% Triton X 100 (*v/v*), 2 mm PMSF, 0.02% (*w/v*) NaN_3_). Cells were lysed on ice for 10 min and nuclear separation was obtained by centrifugation at 6500× *g* for 10 min at 4 °C. Nuclei were then suspended in 0.2 N HCl. After an overnight incubation at 4 °C on a thermomixer, samples were centrifuged and the histone concentration in the supernatant was determined by using the Bradford Assay.

### 4.8. Western Blotting

SDS-PAGE and Western blot analyses were performed using standard protocols.

Protein samples from cytosolic and nuclear extracts were denatured, separated on 7% or 10% polyacrylamide, 0.1% SDS (SDS-PAGE), and electro-transferred onto a nitrocellulose blotting membrane (GE Healthcare, Milan, Italy). Following blocking with 5% skimmed milk in TBST buffer (0.01 m Tris-HCl, pH 8.0, 0.15 m NaCl, and 0.1% Tween 20), the membranes were immunoblotted overnight with different primary antibodies.

Each Ab was used according to the manufacturer’s instructions. After extensively washing with TBST, the primary Abs were detected by the appropriate horseradish peroxidase-conjugated secondary Abs (GE Healthcare) and revealed by chemiluminescence and autoradiography.

### 4.9. Cell Proliferation and Cell Cycle Analyses

For cell proliferation analyses, cells were seeded in octuplicate into 96-well plates at a density of 2.5 × 10^3^ cells per well and the experiment was repeated three times (independent biological replicates). After incubation for 3, 6, 9, and 12 days with either drugs or vehicles, cell proliferation was evaluated using 3-(4,5-dimethylthiazol-2-yl)-2,5-diphenyltetrazolium bromide (MTT) (M6494, Thermofisher Scientific) at a final concentration of 1 mg/mL, according to the manufacturer’s instructions. Absorbance was measured at 570 and 620 nm (background) wavelengths by the VICTOR Multilabel Plate Reader (PerkinElmer, Milan, Italy).

For cell cycle analysis, 10^5^ cells were incubated with increasing concentrations of a compound for 3, 6, 9, or 12 days with ICI 182,780, EPZ004777 or the vehicle, by refreshing every 3 days. All assays were carried out in triplicate.

Cells were then fixed in cold 70% ethanol at 4 °C, treated with RNase A (10 μg/mL) for 10 min at room temperature, and stained with 20 μg/mL Propidium iodide (Sigma-Aldrich) for 30 min. Flow cytometry was performed using a BD FACSVerse™ (BD Biosciences, San Josè, CA) and data processed using the software ModFit LT™ (version 5.0, company, city, country). Results shown were obtained from three independent experiments.

### 4.10. Colony Formation Assay

For each cell line, 5 × 10^3^ cells were seeded in triplicate in 6-well plates and treated for 9 days with different compounds, according to the experimental settings. After incubation, cells were fixed with 4% paraformaldehyde and stained with 0.1% (*w/v*) crystal violet in PBS. For quantitation, colonies were dissolved in 10% acetic acid before absorbance measurement at 595 nm. 

### 4.11. Chromatin Immunoprecipitation (ChIP)

ChIP assays were performed by following the protocols previously detailed [46]. 

Furthermore, 100 µL of equilibrated suspension of Dynabeads M-280 Sheep Anti-Rabbit IgG or Anti-Mouse IgG were incubated overnight at 4 °C with 10 µg of the indicated antibodies for the immunoprecipitations. Purified Rabbit IgG and Mouse IgG antibodies were used as negative controls. Crosslinking was performed with 1% formaldehyde at room temperature for 10 min and the reaction was stopped by adding glycine to a final concentration of 0.125 M. Bead washing, elution, reverse crosslinking, and DNA extraction were then performed as described [48]. DNA concentration was determined by using the Quant-IT DNA Assay Kit-High Sensitivity and a Qubit Fluorimeter (Life Technologies).

For ChIP–qPCR, 2 ng of DNA were used to amplify indicated promoter regions with the following specific primers.

ESR1: Forward primer: TGTGCGCCCTAACCAAAGG

  Reverse primer: TGCTCCCAAAGTAGATAGACCCT

HOXB3: Forward primer: CCACCTGCATAAGGGCAAAAT

  Reverse primer: CTACAGGGCTAGGAATGAGGG

TGM1: Forward primer: TGCCCAACCATTCCTTAGCA

  Reverse primer: CTGCTGTTGAGACTTGCCCA

TWIST2: Forward primer: CGACTGTCCTTACACTGGCG

  Reverse primer: CACAGCTAGTCTGCACCGC

WT1: Forward primer: CGTATACCCTTGCTTTGCACC

  Reverse primer: ATCATGGCCACTCCCCTACC

ChIPs were performed in triplicate and qPCRs were carried out in duplicate in each case.

### 4.12. Drug Combination Analysis

Drug combination analysis was carried out by using Combenefit software [49] and dose-response data. Loewe-selected parameters have quantified an additive/synergic effect between two fixed sub-optimal doses of ICI (10^−8^ and 10^−9^ M) and increasing concentrations of EPZ00477 after 9 days of treatment.

### 4.13. Functional Analyses and Pathway Analyses

The lists of differentially expressed transcripts were submitted to Ingenuity Pathway Software (IPA, Ingenuity System, www.ingenuity.com) and investigation of modulated canonical pathways was carried out. The Circos plot was generated using GOPlot [50]. Gene Set Enrichment Analysis (GSEA) [51] was performed on differentially expressed genes in PEO1 and PEO4 cell lines as follows: all genes were ranked and weighted by their log2 fold-change on ICI treatment and given input to the GSEA Preranked tool, using Hallmark Gene Sets Molecular Signatures Database. Only processes/pathways with FDR ≤0.05 were considered for further analysis.

### 4.14. Statistical Analysis

Statistical analyses were performed using R (version 3.4.4). Error bars represent mean ± SD of independent replicates. Comparisons between two groups were conducted by performing student’s *t* test. Differences were considered as statistically significant at *p* ≤ 0.05.

## 5. Conclusions

In the present study, we provide evidence of a key role of DOT1L in OC, including the functional cooperation between the nuclear receptor ERα and this epigenetic writer in conveying estrogen signaling to modulate the transcription of genes involved in cell proliferation and other key functions of OC cells. Furthermore, the results provide evidence suggesting how combined inhibition of both these factors might be therapeutically effective in blocking estrogen signaling in chemo-resistant ovarian tumors. 

## Supplementary Materials

The following are available online at https://www.mdpi.com/2072-6694/11/11/1720/s1. Figure S1: H3K79 methylation and cell proliferation inhibition by selective DOT1L blockage with EPZ-5676 or SGC0946, Figure S2: Effects of EPZ on ERα-negative OC cell transcriptome, Figure S3: ERα expression analysis following alternative DOT1L inhibitors treatment, Figure S4: effect of EPZ00477 histone transcription markers, Figure S5: ERα and DOT1L binding to EPZ-downregulated genes, Figure S6: DOT1L gene expression correlates with clinical behavior of OC and is an essential gene in a large number of OC cell models, Table S1: PEO cells undergoing differential expression analysis, Table S2: Transcripts differentially regulated in both PEO1 and PEO4 cells by EPZ and ICI showing one or more ERE motifs in the promoter.
