# Supplementary material for: The Histone Methyltransferase DOT1L Is a Functional Component of Estrogen Receptor Alpha Signaling in Ovarian Cancer Cells
Source: Cancers (Basel). 2019 Nov 4;11(11):1720. doi: 10.3390/cancers11111720 (PMC6895927; doi:10.3390/cancers11111720)
Supplement: Supplementary file 1 [file cancers-11-01720-s001.zip › cancers-604602-supplementary-final/Supplementary_files_r2/cancers-604602-suppl-v2.docx]

The Histone Methyltransferase DOT1L is A Functional Component of Estrogen Receptor Alpha Signaling in Ovarian Cancer Cells

Annamaria Salvati, Valerio Gigantino, Giovanni Nassa, Giorgio Giurato, Elena Alexandrova, Francesca Rizzo, Roberta Tarallo and Alessandro Weisz


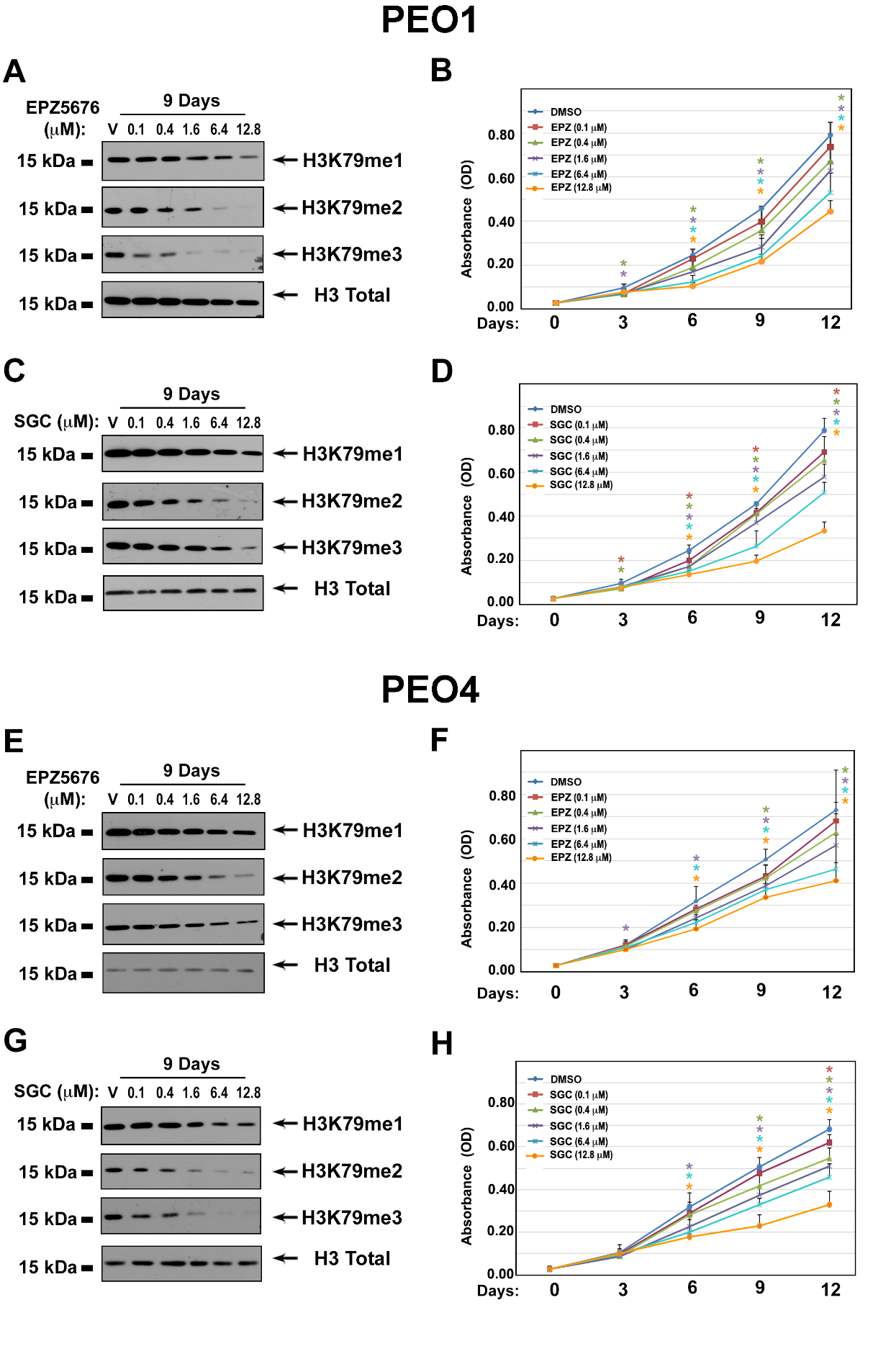


**Figure S1.** H3K79 methylation and cell proliferation inhibition by selective DOT1L blockage with EPZ-5676 or SGC0946. WB showing H3K79me1, H3K79me2 and H3K79me3 levels, compared to total H3, in PEO1 and PEO4 cells following 9 days of treatment with DMSO (V, Vehicle), EPZ5676 (**A** and **E**) or SGC0946 (**C** and **G**) at the indicated concentrations. Cell proliferation rate in presence of increasing concentrations of EPZ5676 (**B** and **F**) and SGC0946 (**D** and **H**), in exponentially growing PEO1 and PEO4 cells, assessed by MTT assay. Error bars represent the mean of replicate values ± SD (*≤0.05).


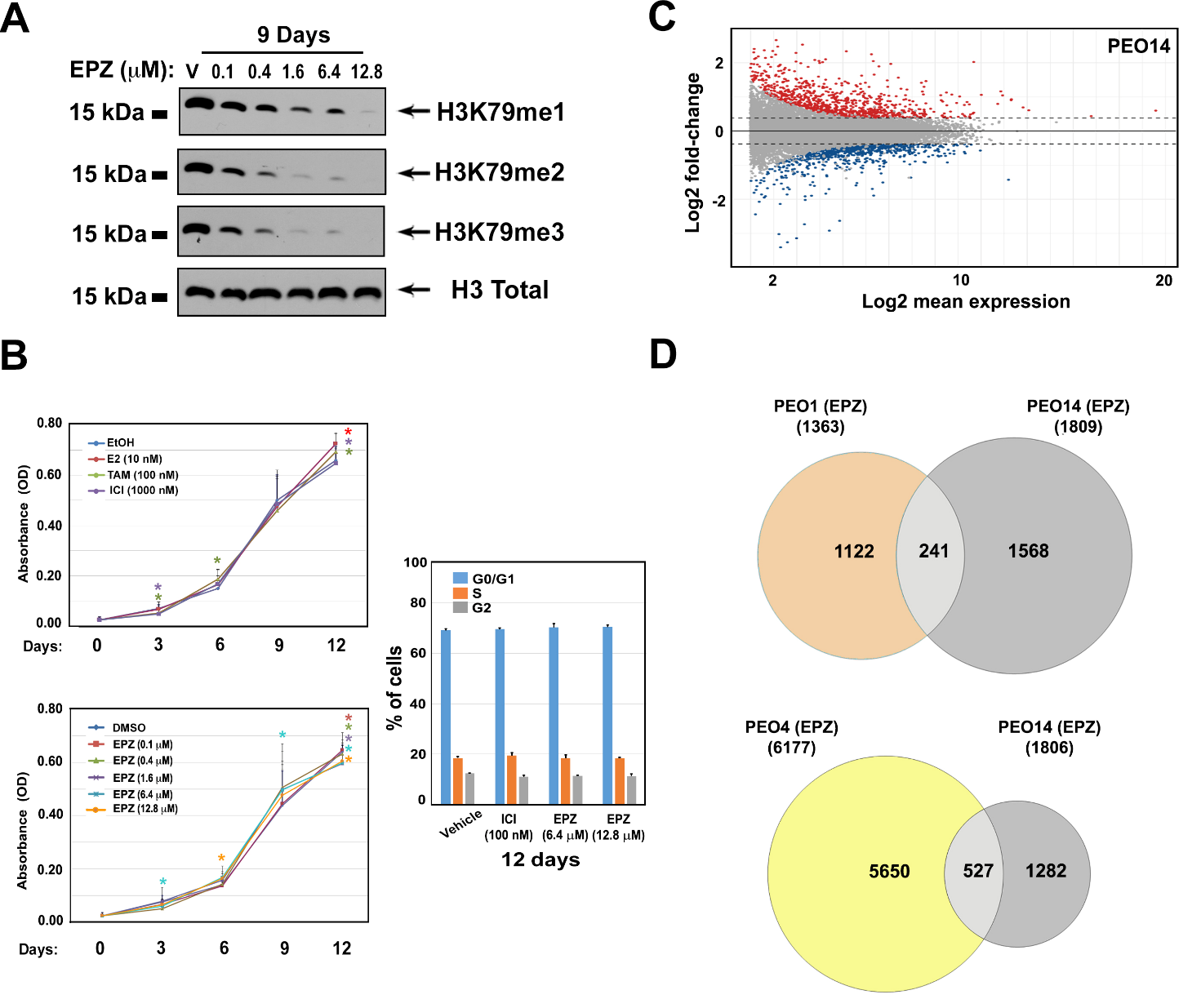


**Figure S2.** Effects of EPZ on ERα-negative OC cell transcriptome. (**A)** Immunoblotting analysis showing effects of EPZ in H3K79me1, H3K79me2 and H3K79me3 levels, compared to total H3, after 9 days of treatment in PEO14 cell line. (**B**) Cell proliferation rate, assessed by MTT assay (left) and cell cycle analysis (right) in presence of antiestrogens or increasing concentrations of EPZ4004777 in exponentially growing PEO14 cells. Error bars represent the mean of replicate values ± SD (*≤0.05). (**C)** MA plot showing genes differentially expressed in PEO14 following 9 days of treatment with EPZ (6.4 μM) *vs* untreated cells. (**D)** Venn diagrams showing common differentially expressed RNA transcripts between PEO14 and PEO1 *(upper panel)* or PEO4 *(down panel)* after EPZ treatment.


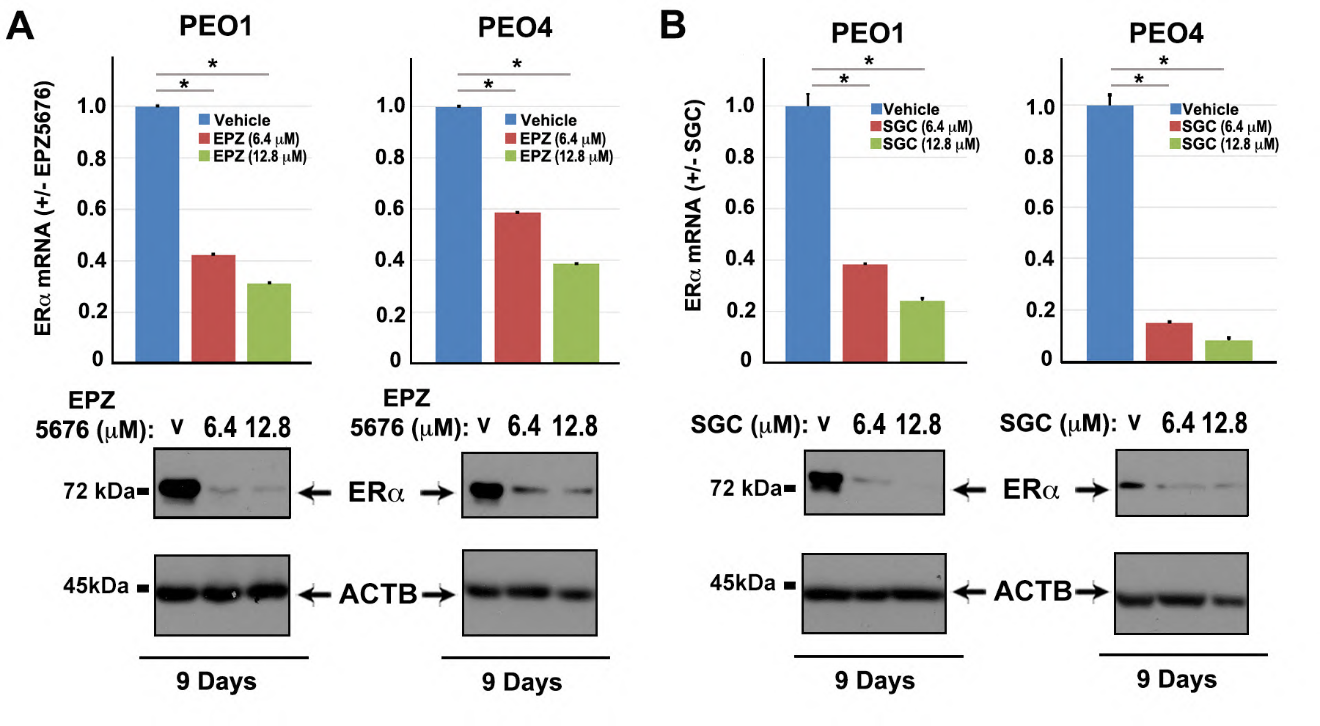


**Figure S3.** ERα expression analysis following alternative DOT1L inhibitors treatment and effect of EPZ00477 histone transcription markers. ERα mRNA and protein levels assessed by RT-qPCR (*upper panels*) and immunobloting analysis (*lower panels*) in PEO1 (*left*) and PEO4 (*right*) treated with EPZ5676 (**A**) and SGC0946 (**B**) at the indicated conditions.


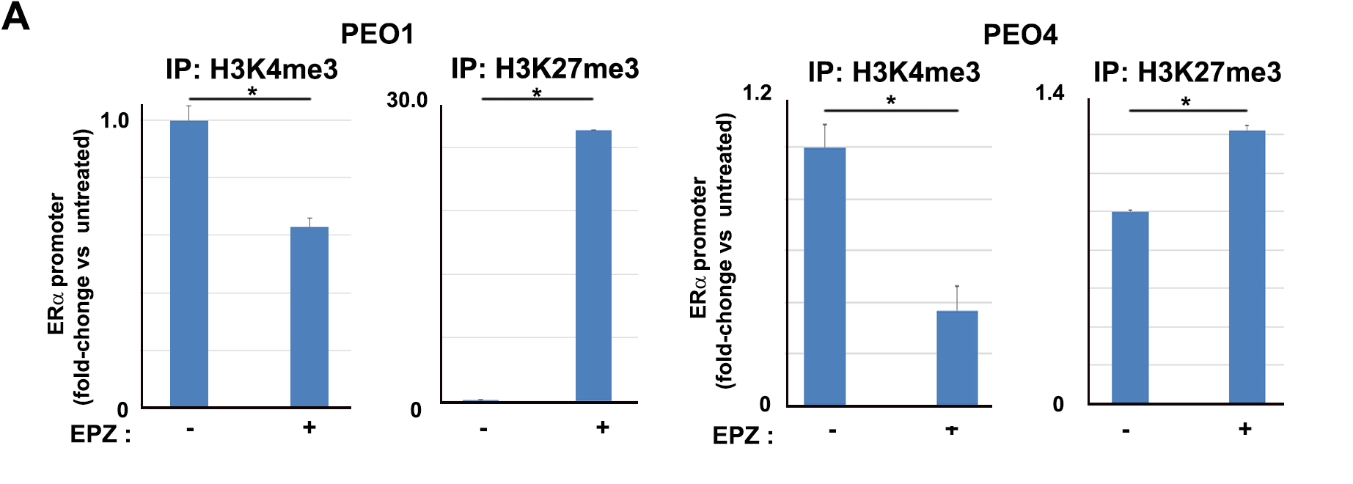


**Figure S4.** ChIP-qPCR showing H3K4me3 and H3K27me3 occupancy within ESR1 promoter region before and after EPZ treatment in PEO1 (*upper panel*) and PEO4 (*lower panel*) cell lines. qPCR data are reported as the mean ± S.D. of independent triplicate measurements (**p* ≤ 0.05).


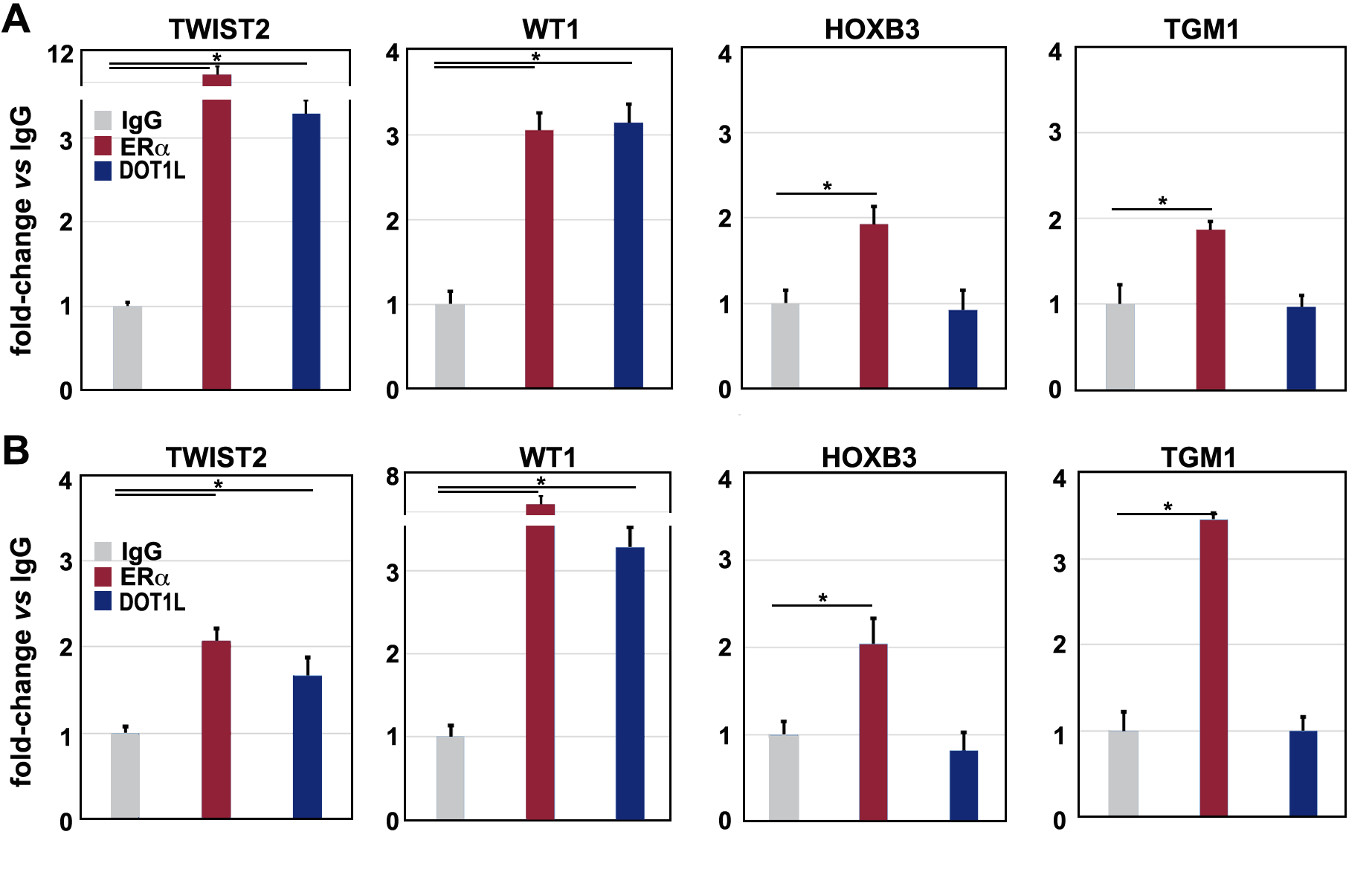


**Figure S5.** ChIP-qPCR showing ERα and DOT1L occupancy within ERE-containing promoter regions of four EPZ-downregulated genes measured in PEO1 (**A**) and PEO4 (**B**) cells. qPCR data are reported as the mean ± S.D. of independent triplicate measurements (**p* ≤0.05).


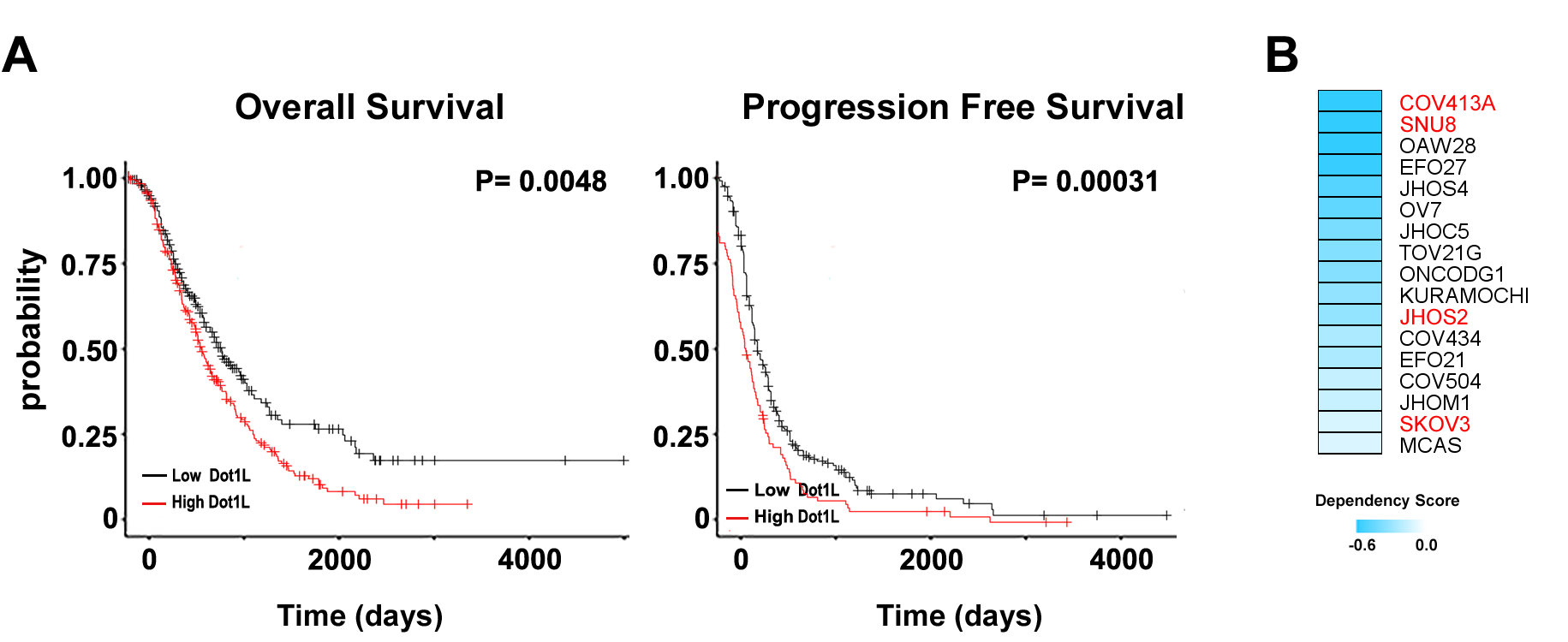


**Figure S6.** DOT1L gene expression correlates with clinical behavior of OC and is an essential gene in a large number of OC cell models. (**A)** Kaplan-Meier curves showing overall survival and progression-free survival for HGSOC patients with high or low DOT1L gene expression level in the tumor. (**B)** Heatmap showing OC cell lines with significantly negative dependency score for DOT1L gene, indicating a higher likelihood that it is an essential gene in the reported cell line, based on data from cell depletion assay (CRISPR AVANA Public 19Q3 dataset) obtained from depmap portal (<https://depmap.org/portal>). In red are highlighted ERα-positive cell lines showing a negative dependency score also for the receptor.

**Whole Western Blots**


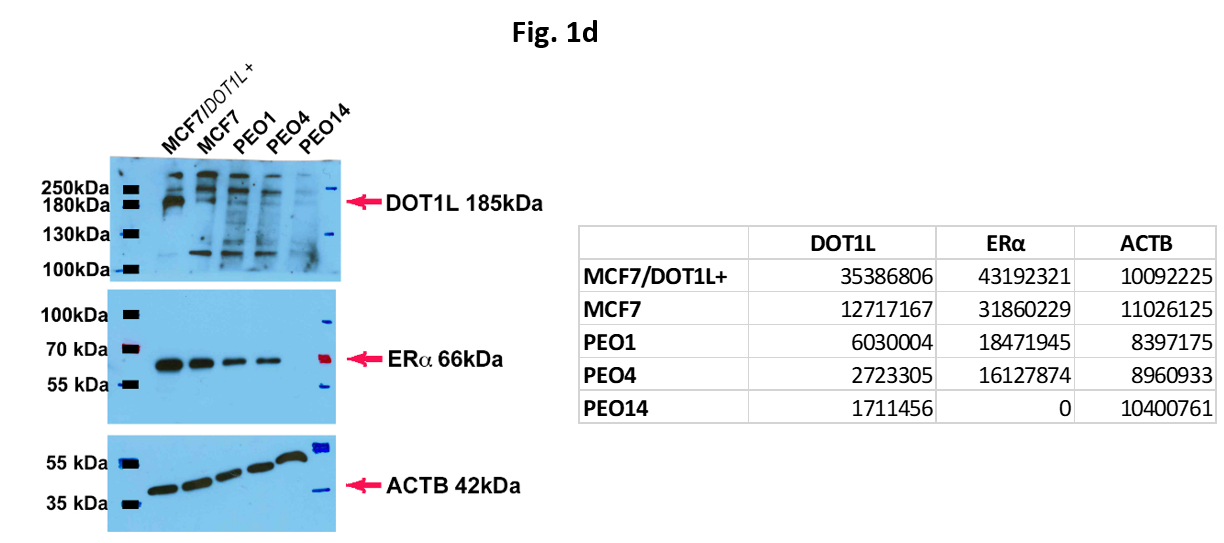


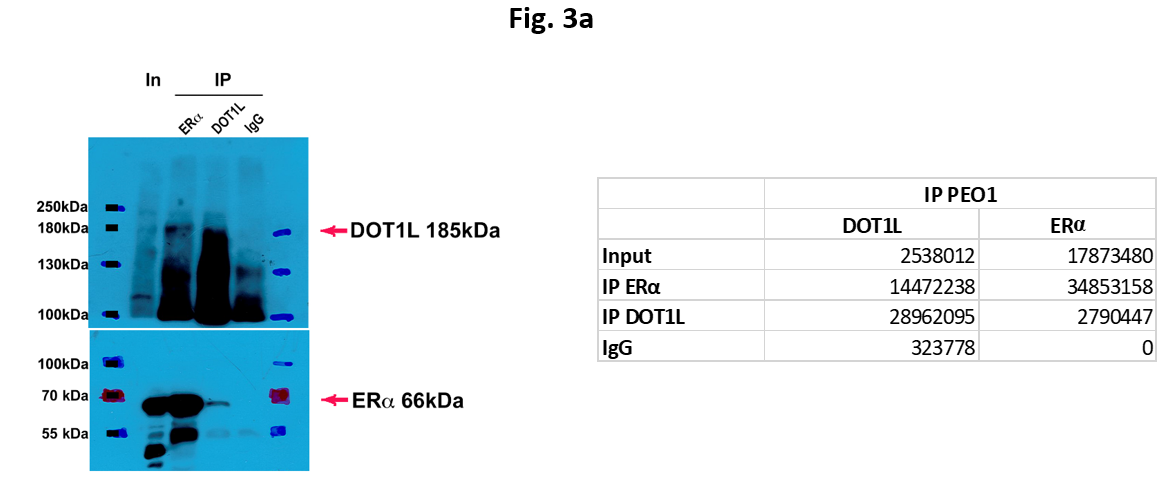


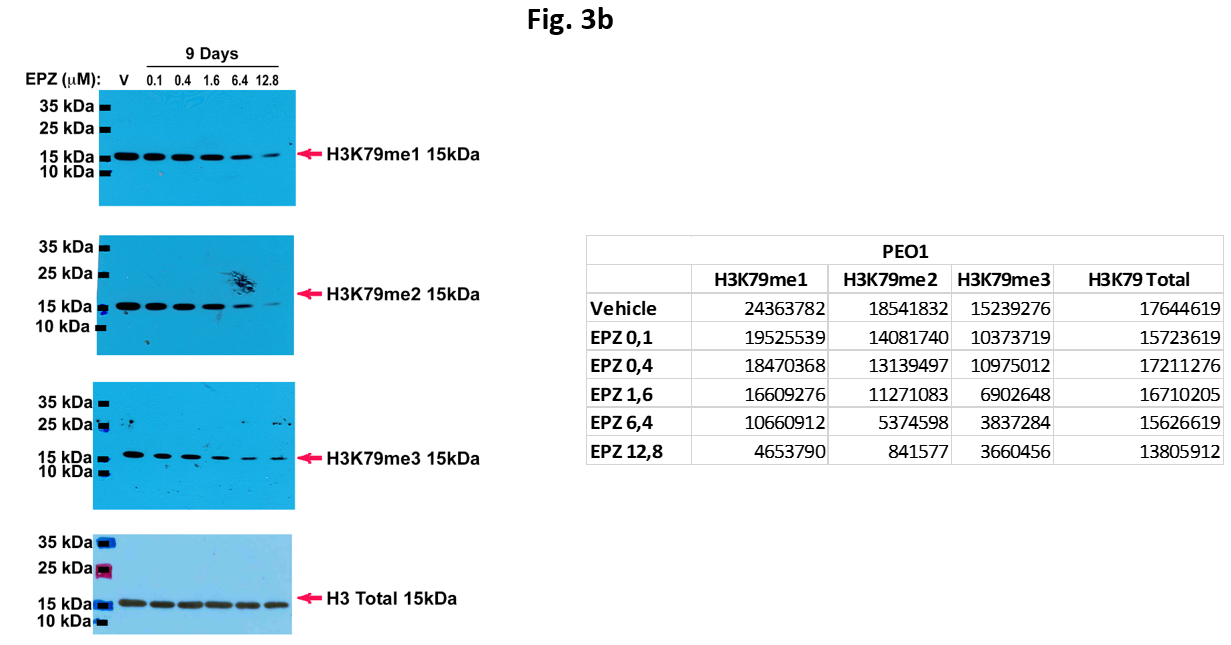


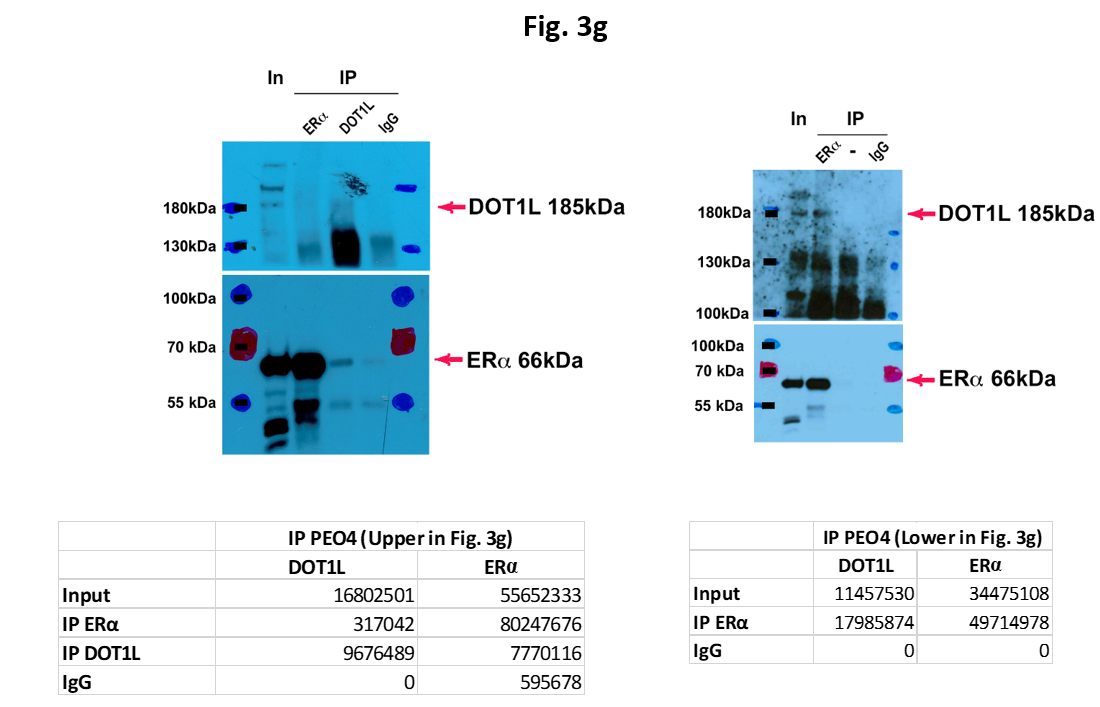


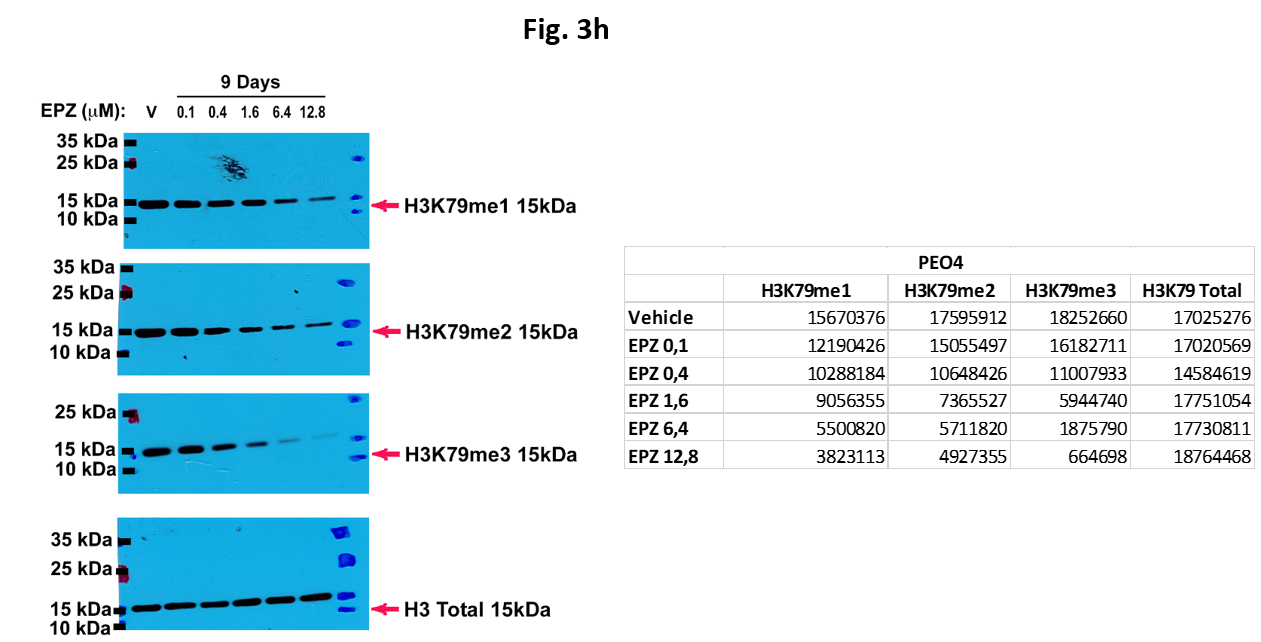


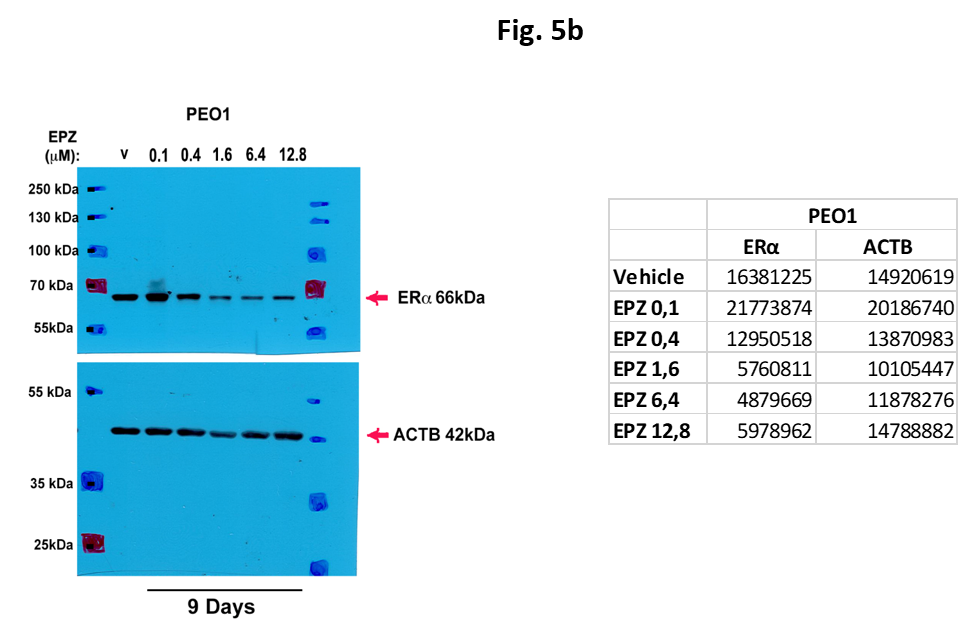


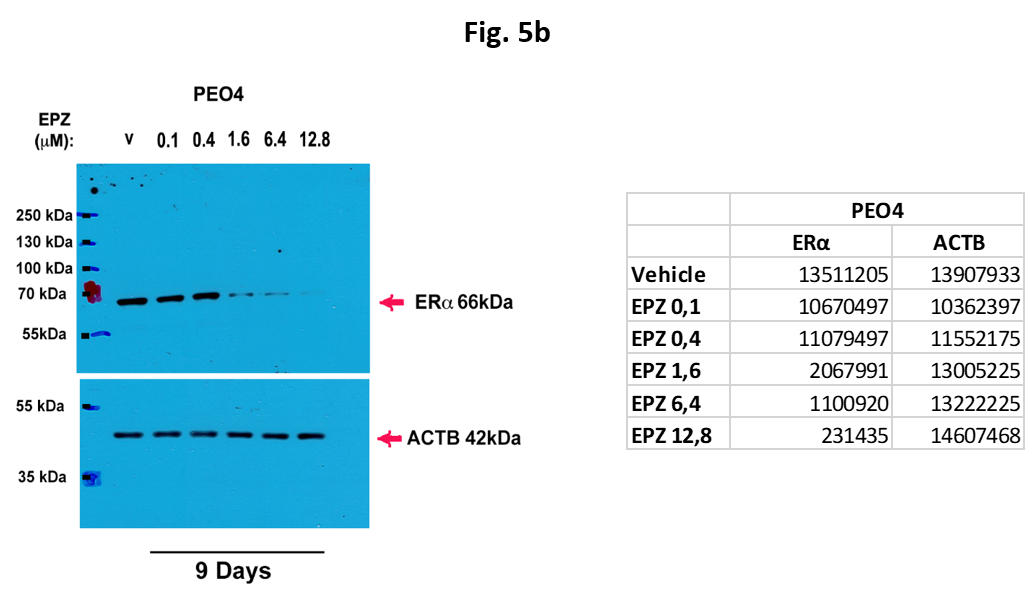


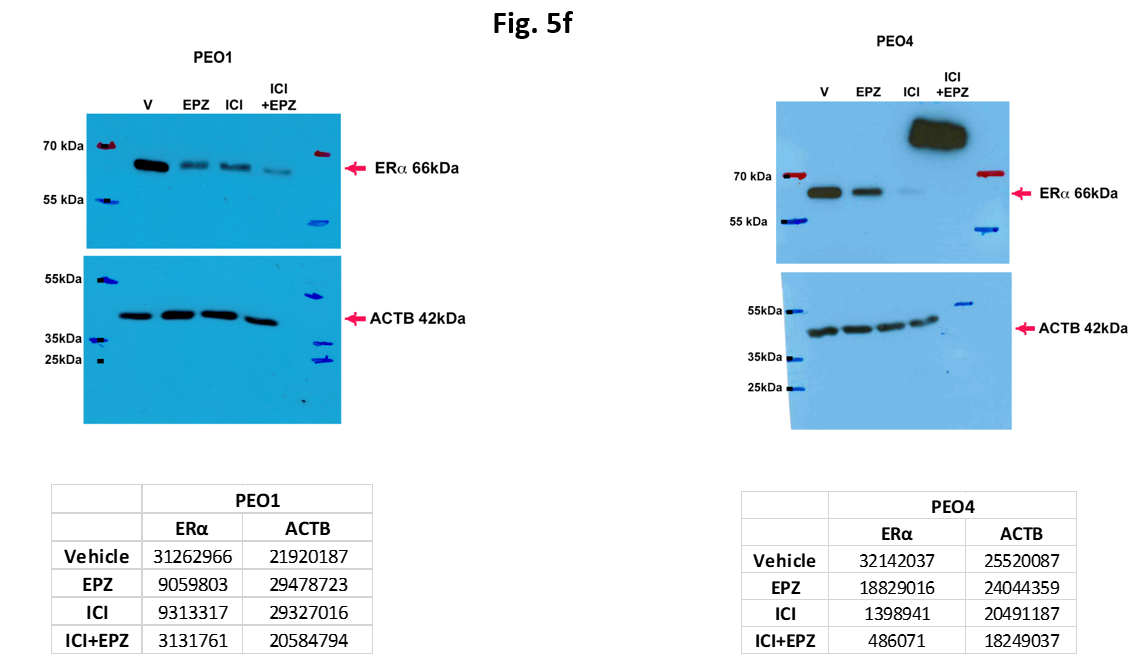


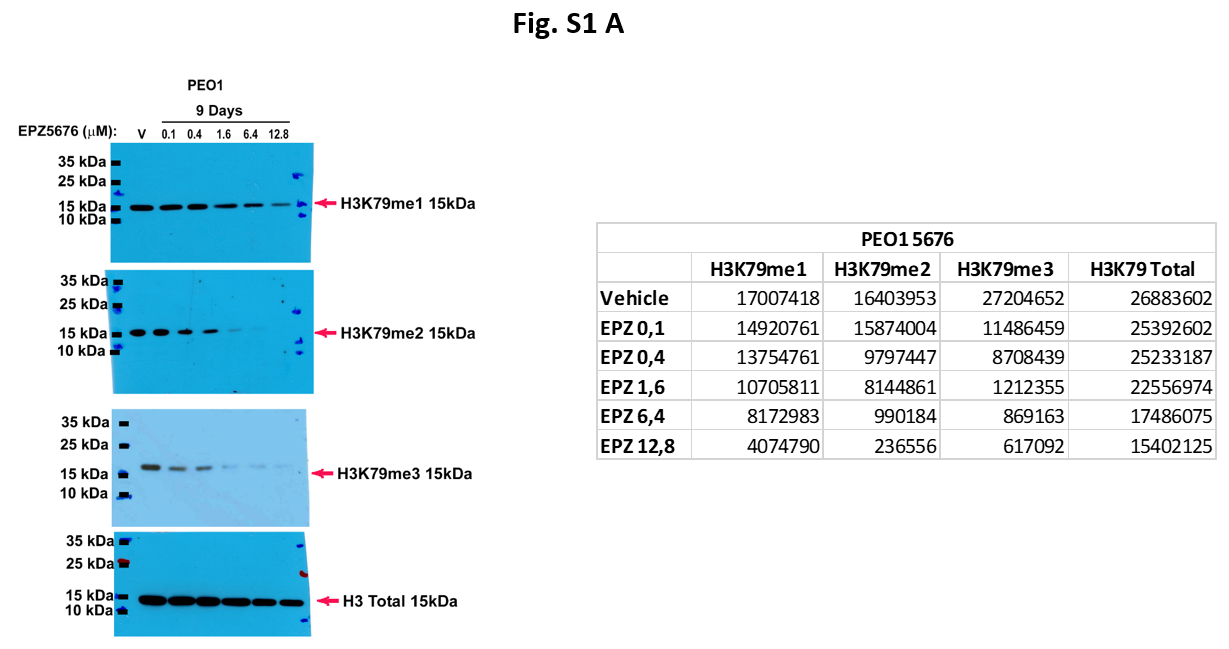


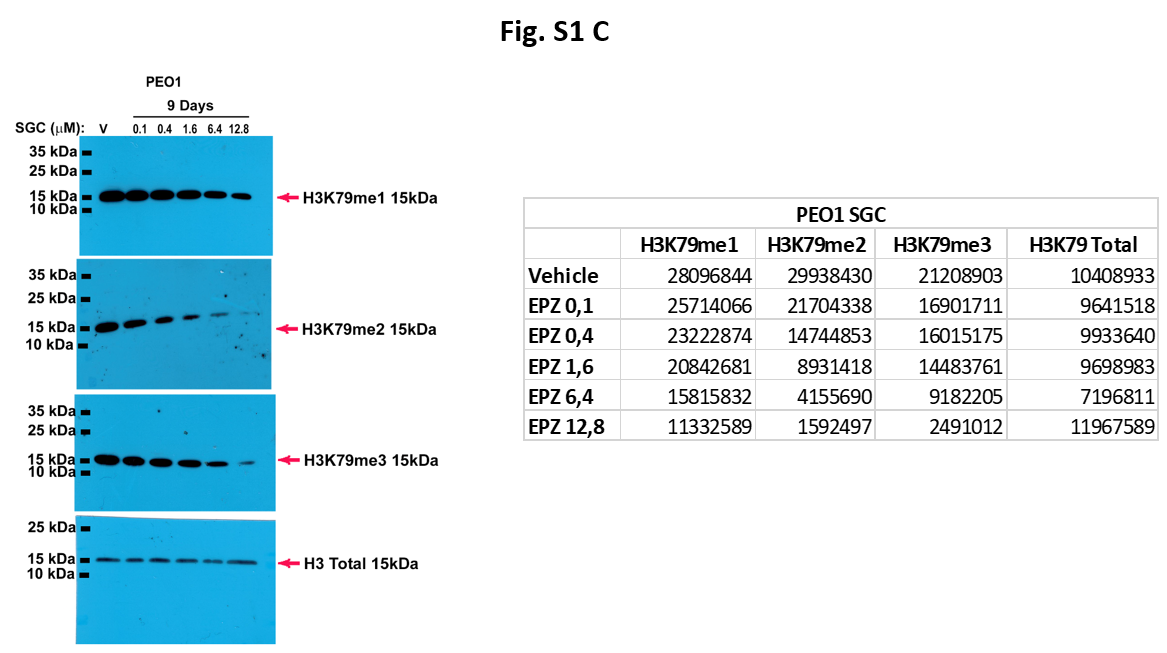


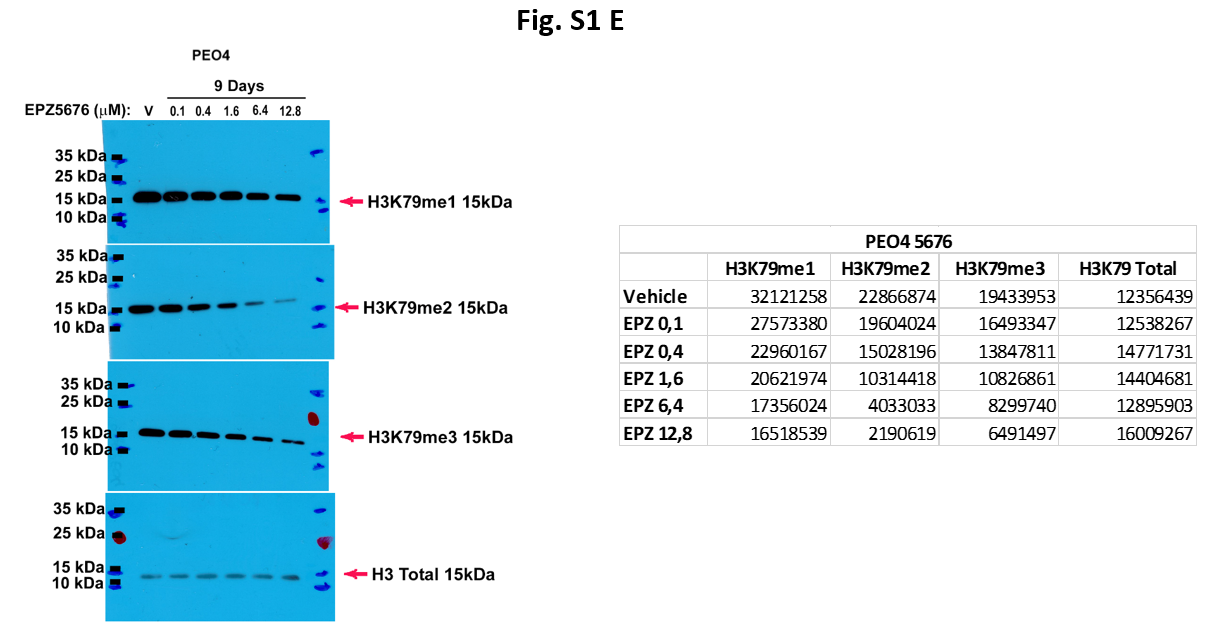


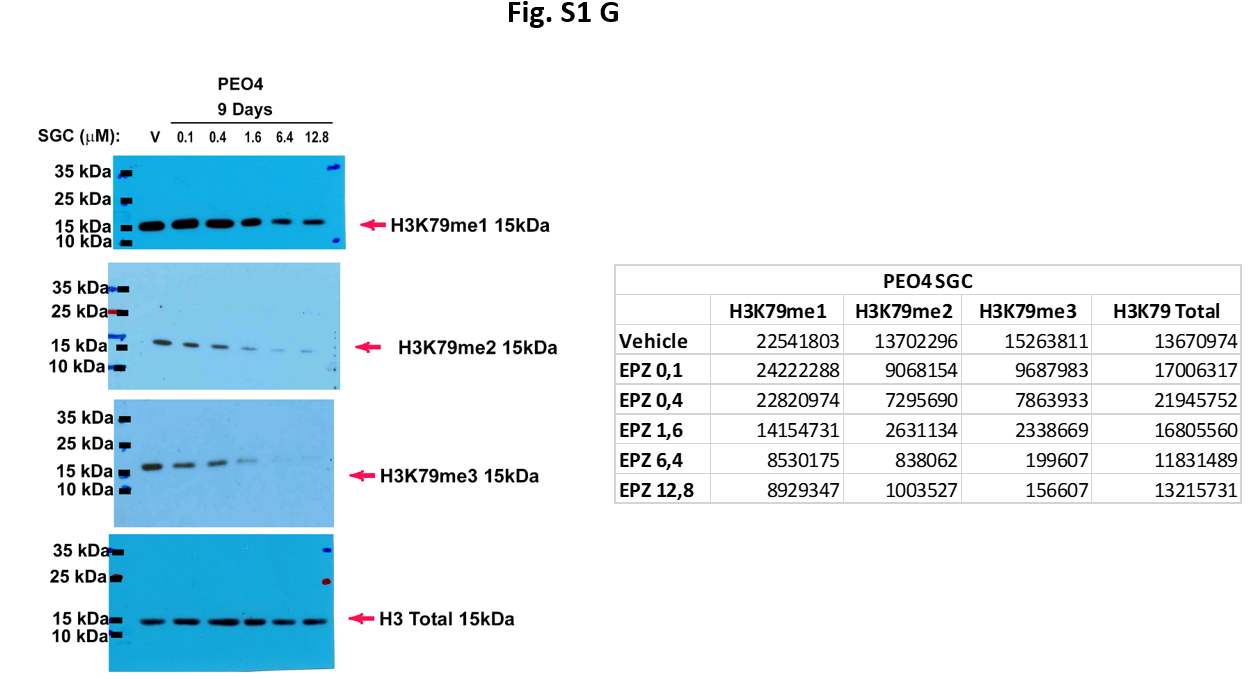


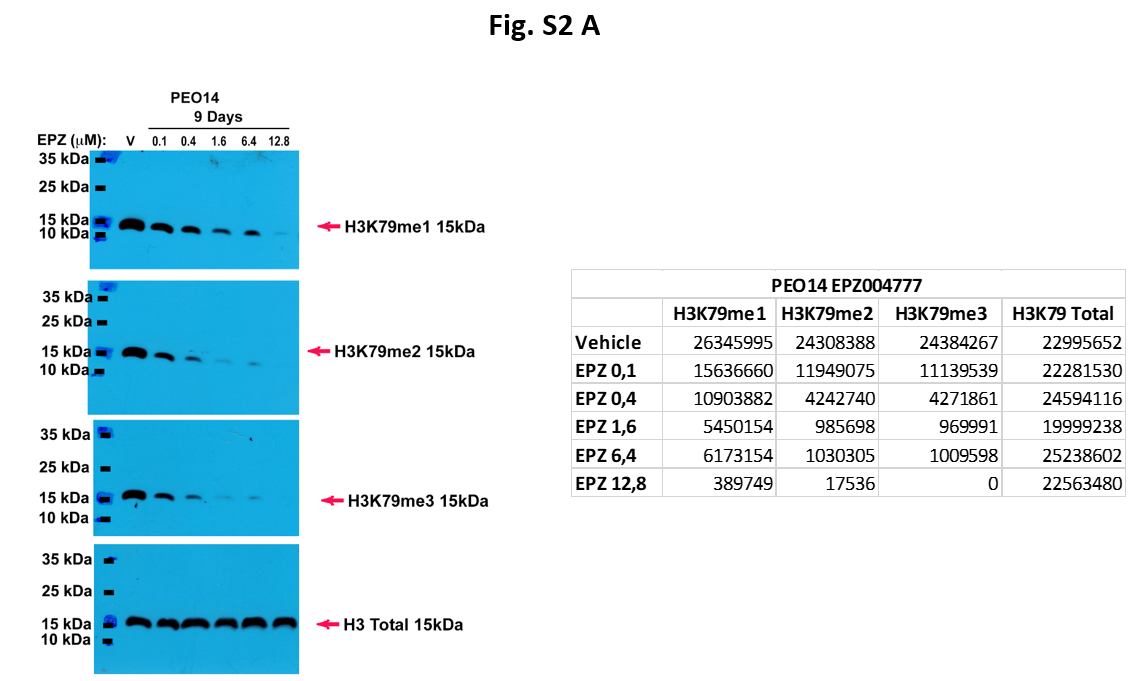


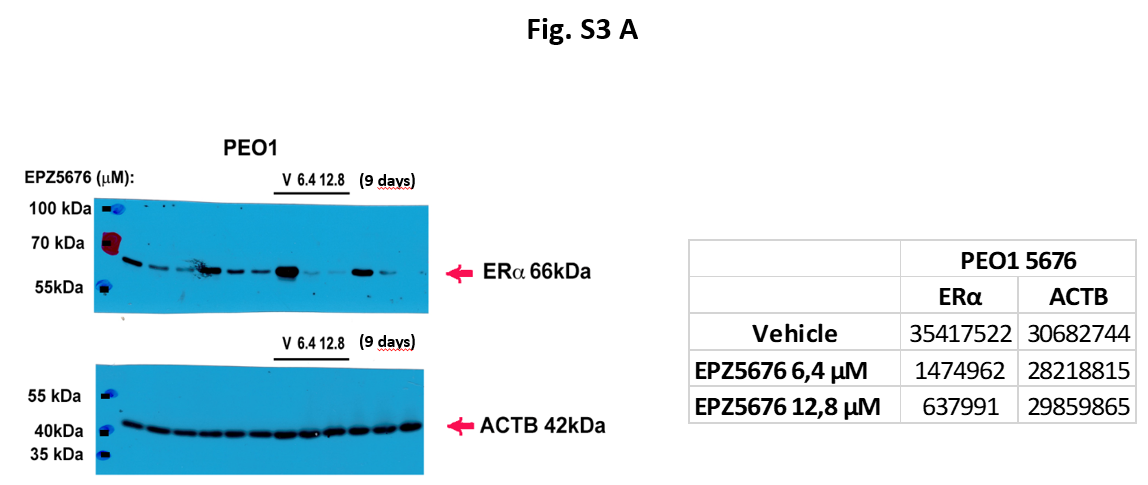


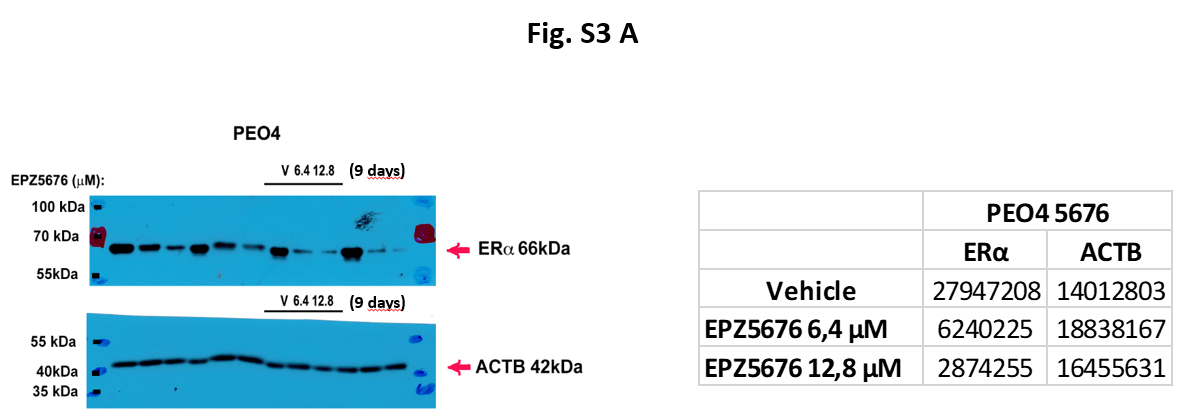


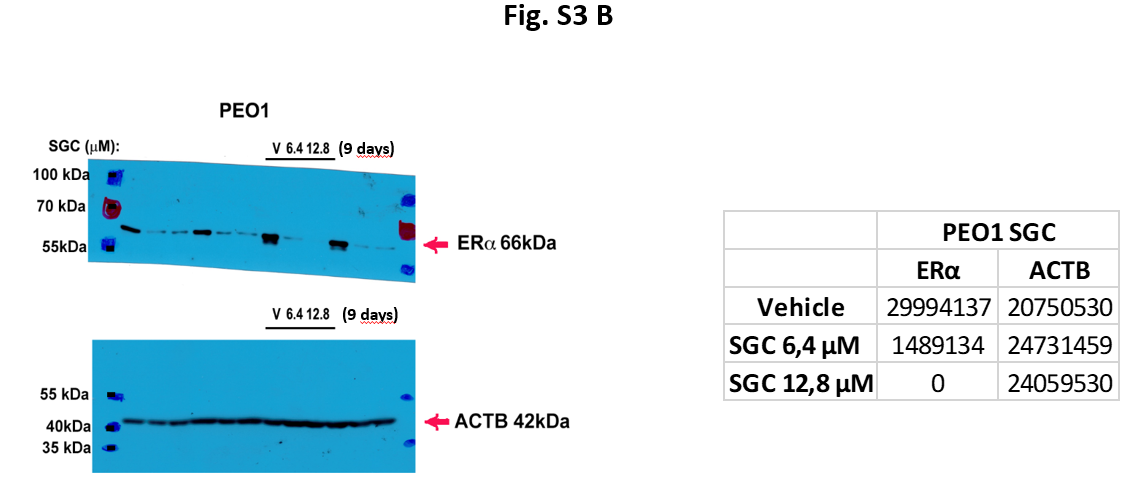


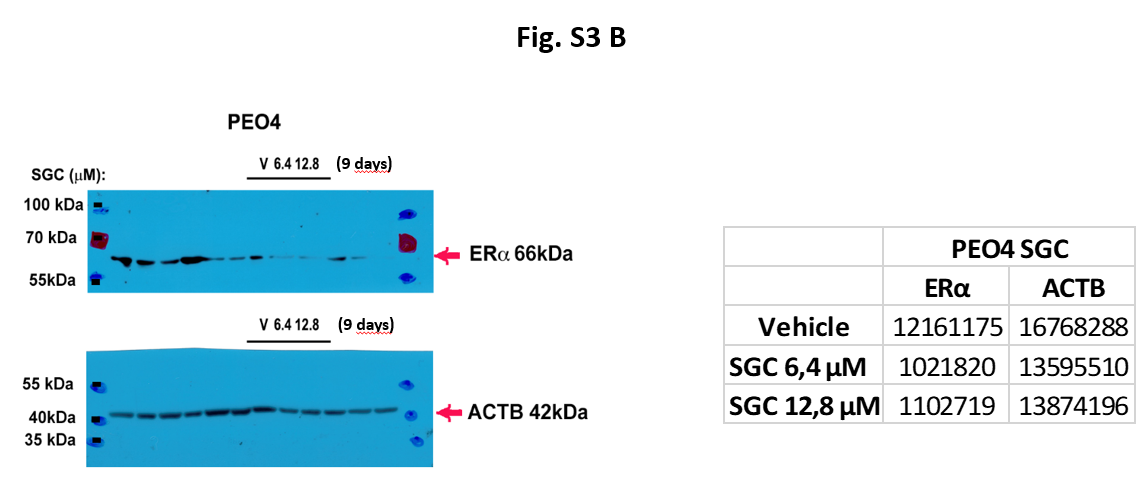


Images were processed with ImageJ software ([https://imagej.net](https://imagej.net/)) for densitometry readings.
